# Supplementary figures and images for: MicroRNA-371–373 cluster extracellular vesicle-based communication in testicular germ cell tumors
Source: Cell Commun Signal. 2025 May 30;23:252. doi: 10.1186/s12964-025-02250-8 (PMC12124061; doi:10.1186/s12964-025-02250-8)

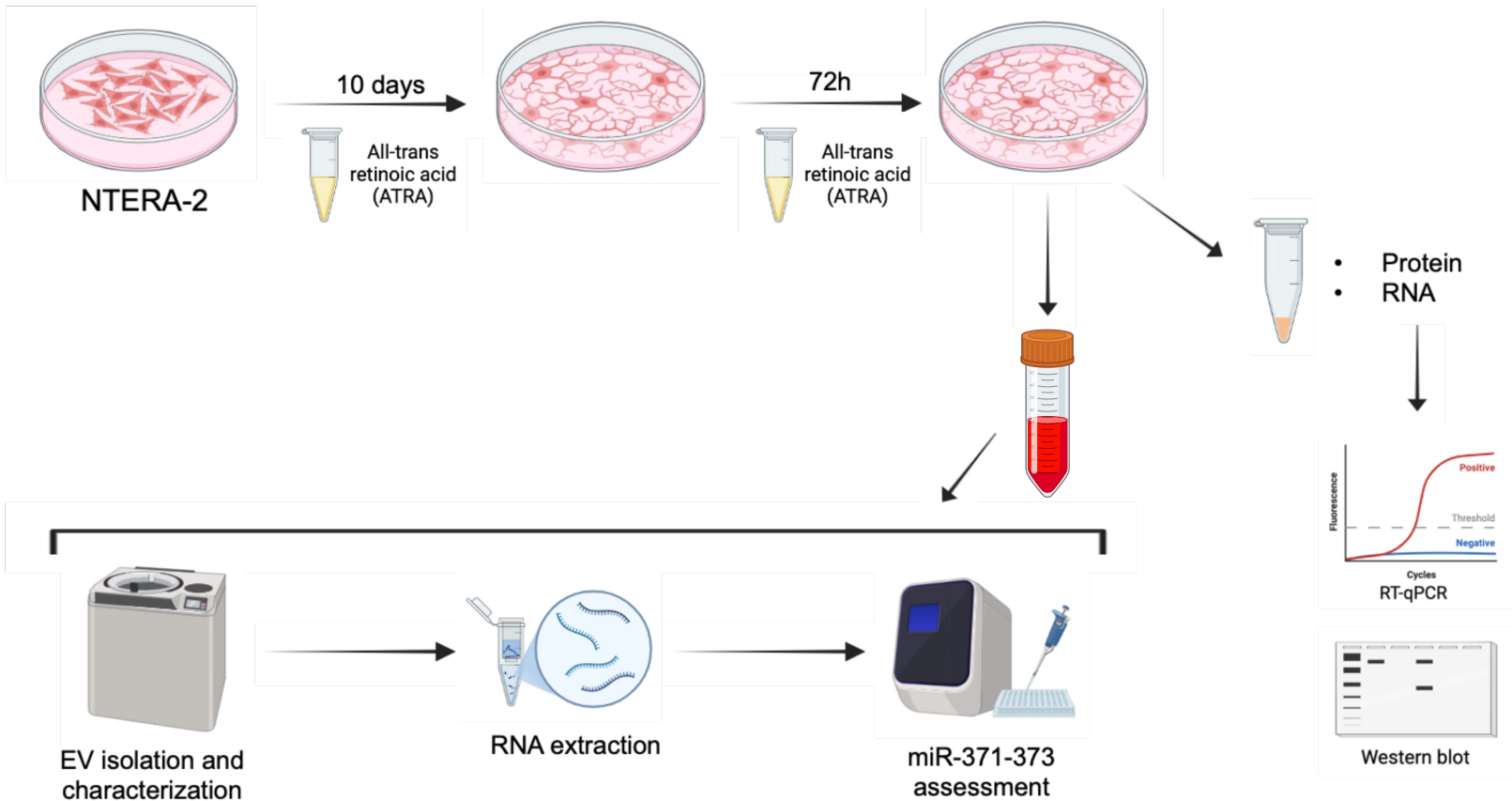

Supplement: Supplementary file 1 — Supplementary Material 1: Figure S1: Graphical representation of ATRA treatment schematic used for in the NT2 cell line. Figure S2: Total number of particles/ml (A, B), and mode particle size (C, D) for all separate (T)GCT cell lines lEV and sEV NTA experiments. Data shown as mean ± SEM for 3 independent experiments. Figure S3: RNA concentration measurement for the (T)GCT cell line-derived EV populations (A) and RNA concentration normalized to the number of particles: RNA per particle ratio (B). Data shown as mean ± SEM; * - p < 0.05. Figure S4: Raw figures of the western blots performed for the cell and tissue-derived EVs. Red arrow pinpoint blots that were represented in Figure 2A. Figure S5: Raw figures of the western blots performed for the plasma-derived EVs. Red arrow pinpoint blots that were represented in Figure 2B. Figure S6: Representative bright field microscopy imaging of NT2 cells 5 and 10 days after treatment start with vehicle (A,B) and ATRA (C,D). Figure S7: Western blot for pluripotency-related factors NANOG and PAX6, and for Beta-actin in vehicle and ATRA-treated NT2 cells. Figure S8: Raw figures of the western blots performed for the ATRA-treated cells. Red arrow pinpoint blots that were represented in Figure S7. Figure S9: Secretion range (NTA particles per cell ratio) for vehicle and ATRA-treated cells, in lEV (A) and sEV (B) populations. Data shown as mean ± SEM for 5 independent experiments; * - p < 0.05. Figure S10: RNA concentration measurements in tumor tissue vs non-tumoral adjacent tissue, in lEV (A) and sEV (B) populations. Data shown as mean ± SEM for 5 independent experiments; ** - p < 0.01. Figure S11: Tissue cellular levels for miR-371a-3p (A), miR-372-3p (B), miR-373-3p (C) and let-7e (D), in non-TE TGCT, TE and adjacent testicular parenchyma tissues. Data shown on a log scale as mean ± SEM; * - p < 0.05, *** -p < 0.001. Figure S12: Spearman correlation analysis for tissue cellular and conditioned medium (CM) lEV and sEV microRNA le [file 12964_2025_2250_MOESM1_ESM.zip › FigureS1.pdf]

IEV

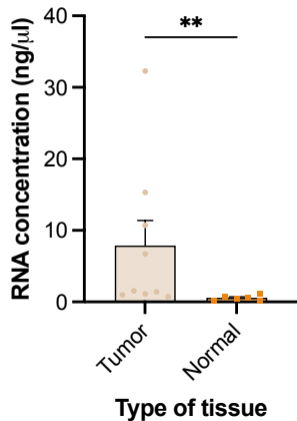

sEV

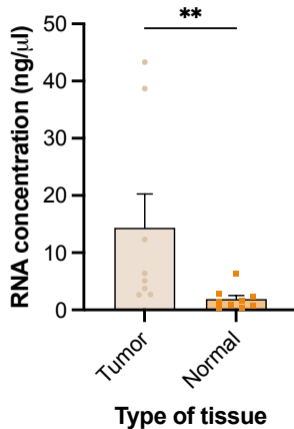

Supplement: Supplementary file 1 — Supplementary Material 1: Figure S1: Graphical representation of ATRA treatment schematic used for in the NT2 cell line. Figure S2: Total number of particles/ml (A, B), and mode particle size (C, D) for all separate (T)GCT cell lines lEV and sEV NTA experiments. Data shown as mean ± SEM for 3 independent experiments. Figure S3: RNA concentration measurement for the (T)GCT cell line-derived EV populations (A) and RNA concentration normalized to the number of particles: RNA per particle ratio (B). Data shown as mean ± SEM; * - p < 0.05. Figure S4: Raw figures of the western blots performed for the cell and tissue-derived EVs. Red arrow pinpoint blots that were represented in Figure 2A. Figure S5: Raw figures of the western blots performed for the plasma-derived EVs. Red arrow pinpoint blots that were represented in Figure 2B. Figure S6: Representative bright field microscopy imaging of NT2 cells 5 and 10 days after treatment start with vehicle (A,B) and ATRA (C,D). Figure S7: Western blot for pluripotency-related factors NANOG and PAX6, and for Beta-actin in vehicle and ATRA-treated NT2 cells. Figure S8: Raw figures of the western blots performed for the ATRA-treated cells. Red arrow pinpoint blots that were represented in Figure S7. Figure S9: Secretion range (NTA particles per cell ratio) for vehicle and ATRA-treated cells, in lEV (A) and sEV (B) populations. Data shown as mean ± SEM for 5 independent experiments; * - p < 0.05. Figure S10: RNA concentration measurements in tumor tissue vs non-tumoral adjacent tissue, in lEV (A) and sEV (B) populations. Data shown as mean ± SEM for 5 independent experiments; ** - p < 0.01. Figure S11: Tissue cellular levels for miR-371a-3p (A), miR-372-3p (B), miR-373-3p (C) and let-7e (D), in non-TE TGCT, TE and adjacent testicular parenchyma tissues. Data shown on a log scale as mean ± SEM; * - p < 0.05, *** -p < 0.001. Figure S12: Spearman correlation analysis for tissue cellular and conditioned medium (CM) lEV and sEV microRNA le [file 12964_2025_2250_MOESM1_ESM.zip › FigureS10.pdf]

**A**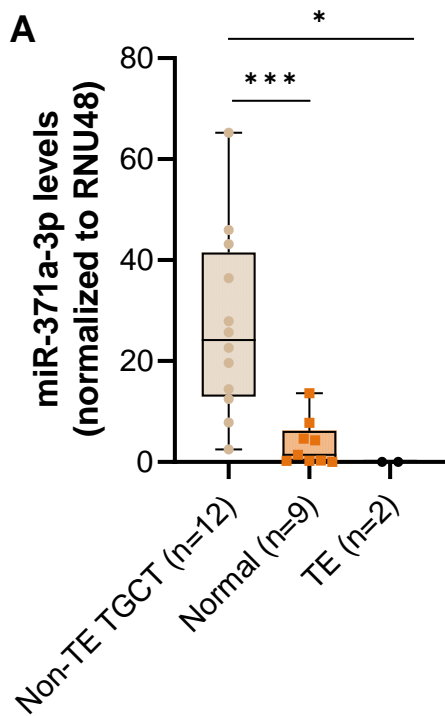**B**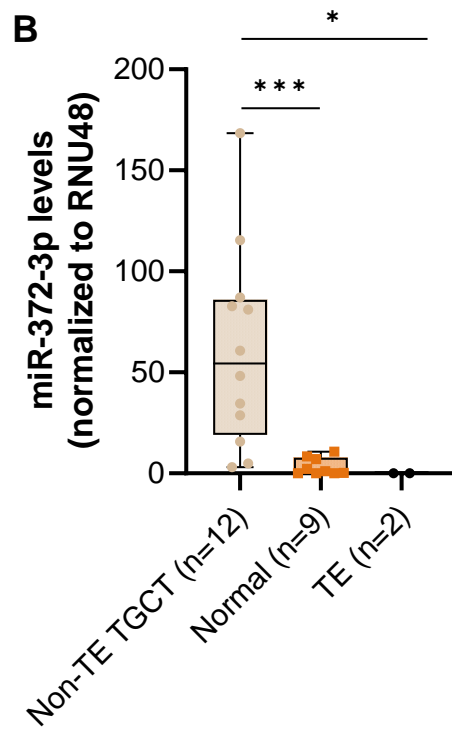**C**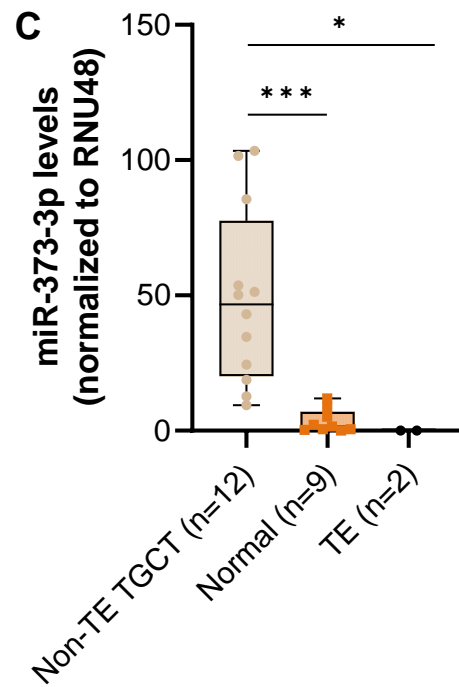**D**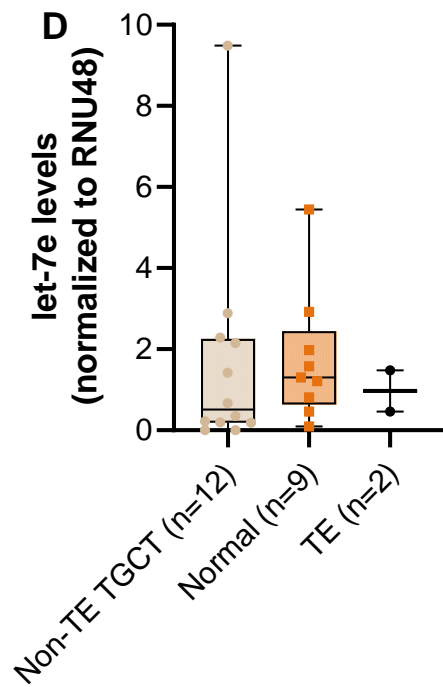

Supplement: Supplementary file 1 — Supplementary Material 1: Figure S1: Graphical representation of ATRA treatment schematic used for in the NT2 cell line. Figure S2: Total number of particles/ml (A, B), and mode particle size (C, D) for all separate (T)GCT cell lines lEV and sEV NTA experiments. Data shown as mean ± SEM for 3 independent experiments. Figure S3: RNA concentration measurement for the (T)GCT cell line-derived EV populations (A) and RNA concentration normalized to the number of particles: RNA per particle ratio (B). Data shown as mean ± SEM; * - p < 0.05. Figure S4: Raw figures of the western blots performed for the cell and tissue-derived EVs. Red arrow pinpoint blots that were represented in Figure 2A. Figure S5: Raw figures of the western blots performed for the plasma-derived EVs. Red arrow pinpoint blots that were represented in Figure 2B. Figure S6: Representative bright field microscopy imaging of NT2 cells 5 and 10 days after treatment start with vehicle (A,B) and ATRA (C,D). Figure S7: Western blot for pluripotency-related factors NANOG and PAX6, and for Beta-actin in vehicle and ATRA-treated NT2 cells. Figure S8: Raw figures of the western blots performed for the ATRA-treated cells. Red arrow pinpoint blots that were represented in Figure S7. Figure S9: Secretion range (NTA particles per cell ratio) for vehicle and ATRA-treated cells, in lEV (A) and sEV (B) populations. Data shown as mean ± SEM for 5 independent experiments; * - p < 0.05. Figure S10: RNA concentration measurements in tumor tissue vs non-tumoral adjacent tissue, in lEV (A) and sEV (B) populations. Data shown as mean ± SEM for 5 independent experiments; ** - p < 0.01. Figure S11: Tissue cellular levels for miR-371a-3p (A), miR-372-3p (B), miR-373-3p (C) and let-7e (D), in non-TE TGCT, TE and adjacent testicular parenchyma tissues. Data shown on a log scale as mean ± SEM; * - p < 0.05, *** -p < 0.001. Figure S12: Spearman correlation analysis for tissue cellular and conditioned medium (CM) lEV and sEV microRNA le [file 12964_2025_2250_MOESM1_ESM.zip › FigureS11.pdf]

**A**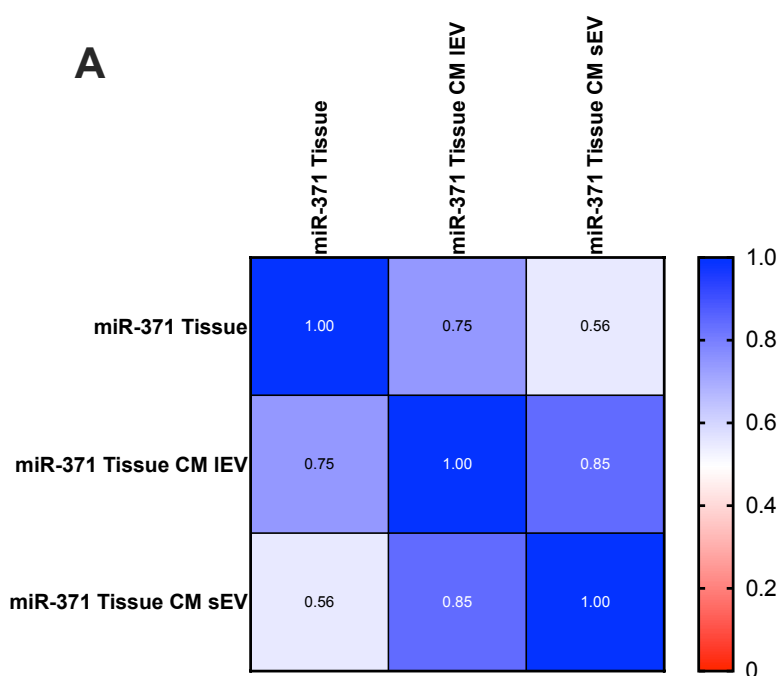**B**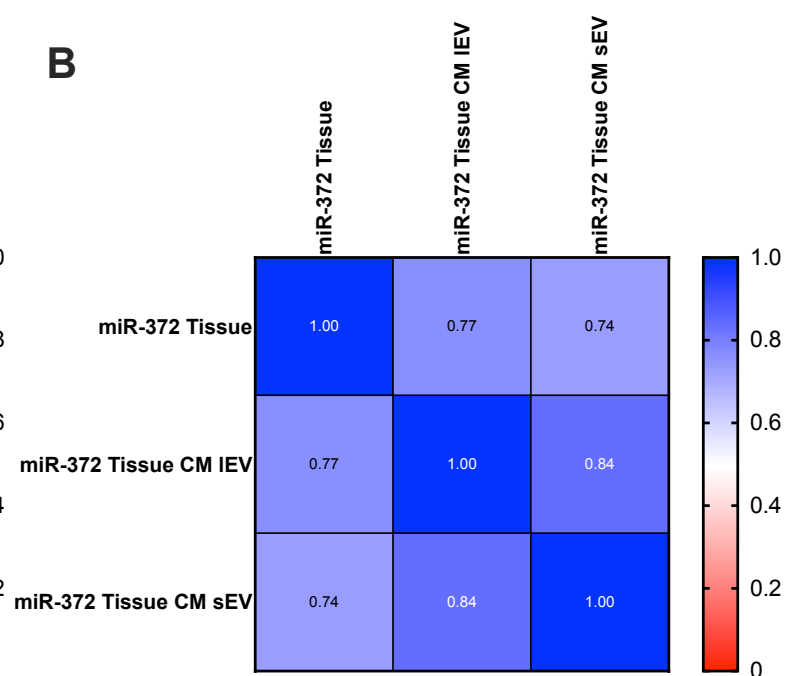**C**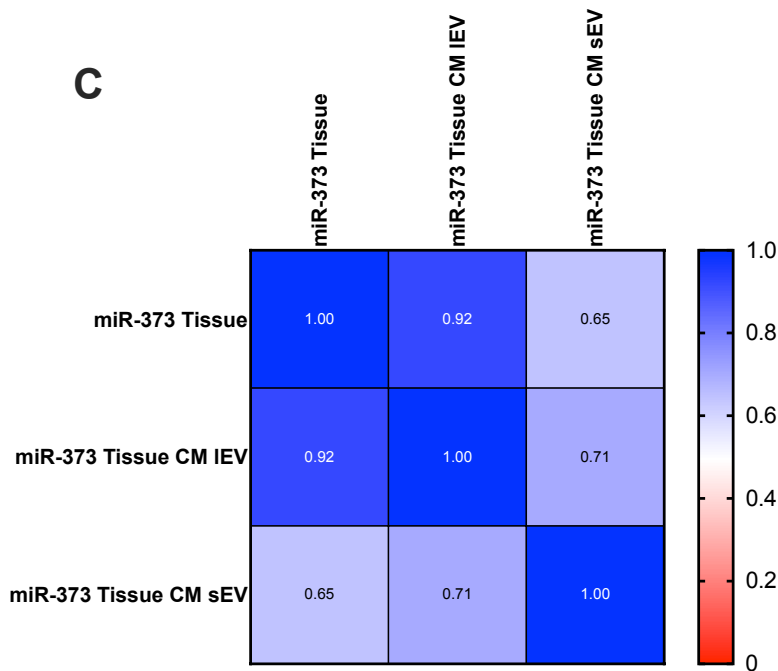**D**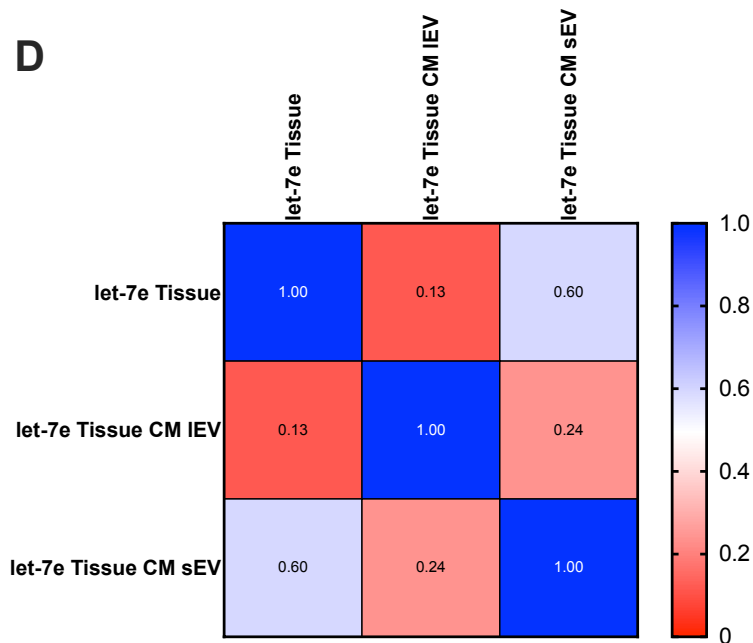

Supplement: Supplementary file 1 — Supplementary Material 1: Figure S1: Graphical representation of ATRA treatment schematic used for in the NT2 cell line. Figure S2: Total number of particles/ml (A, B), and mode particle size (C, D) for all separate (T)GCT cell lines lEV and sEV NTA experiments. Data shown as mean ± SEM for 3 independent experiments. Figure S3: RNA concentration measurement for the (T)GCT cell line-derived EV populations (A) and RNA concentration normalized to the number of particles: RNA per particle ratio (B). Data shown as mean ± SEM; * - p < 0.05. Figure S4: Raw figures of the western blots performed for the cell and tissue-derived EVs. Red arrow pinpoint blots that were represented in Figure 2A. Figure S5: Raw figures of the western blots performed for the plasma-derived EVs. Red arrow pinpoint blots that were represented in Figure 2B. Figure S6: Representative bright field microscopy imaging of NT2 cells 5 and 10 days after treatment start with vehicle (A,B) and ATRA (C,D). Figure S7: Western blot for pluripotency-related factors NANOG and PAX6, and for Beta-actin in vehicle and ATRA-treated NT2 cells. Figure S8: Raw figures of the western blots performed for the ATRA-treated cells. Red arrow pinpoint blots that were represented in Figure S7. Figure S9: Secretion range (NTA particles per cell ratio) for vehicle and ATRA-treated cells, in lEV (A) and sEV (B) populations. Data shown as mean ± SEM for 5 independent experiments; * - p < 0.05. Figure S10: RNA concentration measurements in tumor tissue vs non-tumoral adjacent tissue, in lEV (A) and sEV (B) populations. Data shown as mean ± SEM for 5 independent experiments; ** - p < 0.01. Figure S11: Tissue cellular levels for miR-371a-3p (A), miR-372-3p (B), miR-373-3p (C) and let-7e (D), in non-TE TGCT, TE and adjacent testicular parenchyma tissues. Data shown on a log scale as mean ± SEM; * - p < 0.05, *** -p < 0.001. Figure S12: Spearman correlation analysis for tissue cellular and conditioned medium (CM) lEV and sEV microRNA le [file 12964_2025_2250_MOESM1_ESM.zip › FigureS12.pdf]

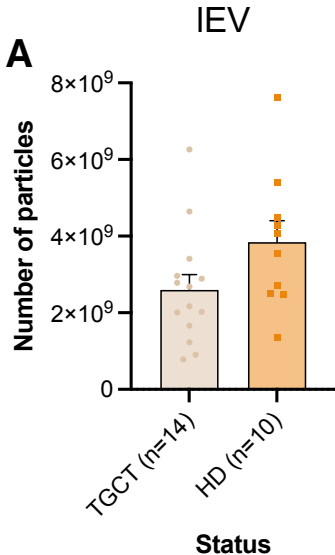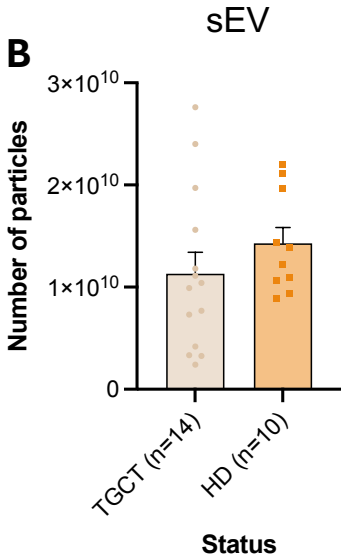

Supplement: Supplementary file 1 — Supplementary Material 1: Figure S1: Graphical representation of ATRA treatment schematic used for in the NT2 cell line. Figure S2: Total number of particles/ml (A, B), and mode particle size (C, D) for all separate (T)GCT cell lines lEV and sEV NTA experiments. Data shown as mean ± SEM for 3 independent experiments. Figure S3: RNA concentration measurement for the (T)GCT cell line-derived EV populations (A) and RNA concentration normalized to the number of particles: RNA per particle ratio (B). Data shown as mean ± SEM; * - p < 0.05. Figure S4: Raw figures of the western blots performed for the cell and tissue-derived EVs. Red arrow pinpoint blots that were represented in Figure 2A. Figure S5: Raw figures of the western blots performed for the plasma-derived EVs. Red arrow pinpoint blots that were represented in Figure 2B. Figure S6: Representative bright field microscopy imaging of NT2 cells 5 and 10 days after treatment start with vehicle (A,B) and ATRA (C,D). Figure S7: Western blot for pluripotency-related factors NANOG and PAX6, and for Beta-actin in vehicle and ATRA-treated NT2 cells. Figure S8: Raw figures of the western blots performed for the ATRA-treated cells. Red arrow pinpoint blots that were represented in Figure S7. Figure S9: Secretion range (NTA particles per cell ratio) for vehicle and ATRA-treated cells, in lEV (A) and sEV (B) populations. Data shown as mean ± SEM for 5 independent experiments; * - p < 0.05. Figure S10: RNA concentration measurements in tumor tissue vs non-tumoral adjacent tissue, in lEV (A) and sEV (B) populations. Data shown as mean ± SEM for 5 independent experiments; ** - p < 0.01. Figure S11: Tissue cellular levels for miR-371a-3p (A), miR-372-3p (B), miR-373-3p (C) and let-7e (D), in non-TE TGCT, TE and adjacent testicular parenchyma tissues. Data shown on a log scale as mean ± SEM; * - p < 0.05, *** -p < 0.001. Figure S12: Spearman correlation analysis for tissue cellular and conditioned medium (CM) lEV and sEV microRNA le [file 12964_2025_2250_MOESM1_ESM.zip › FigureS13.pdf]

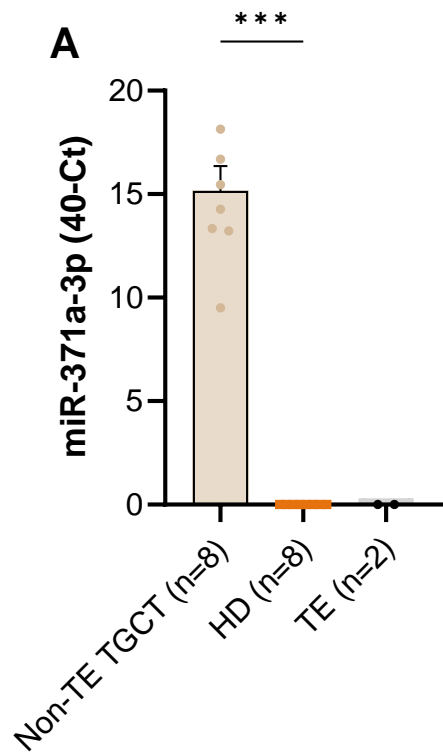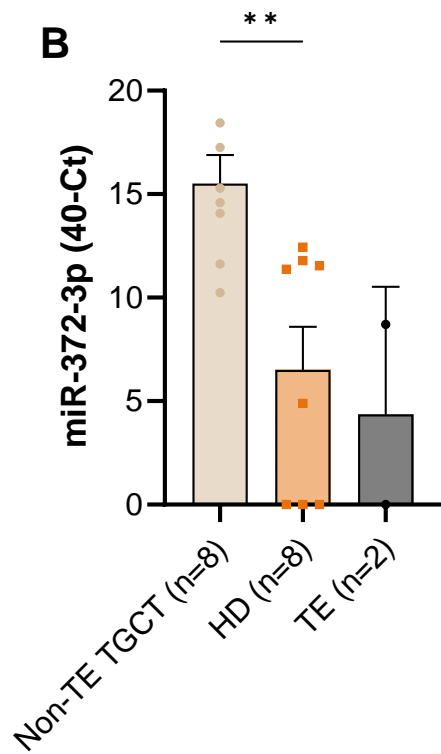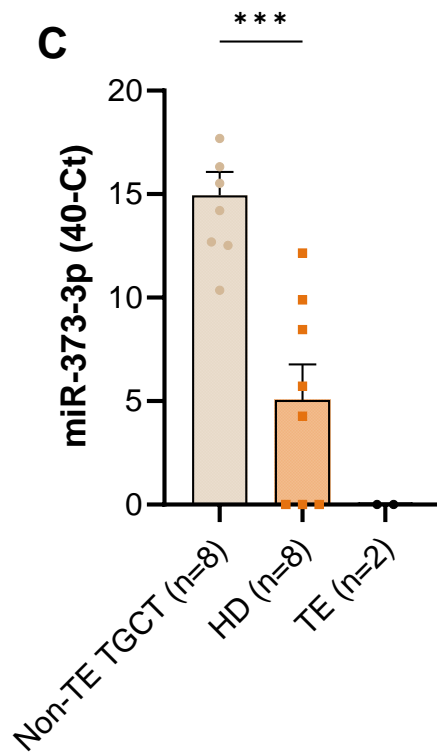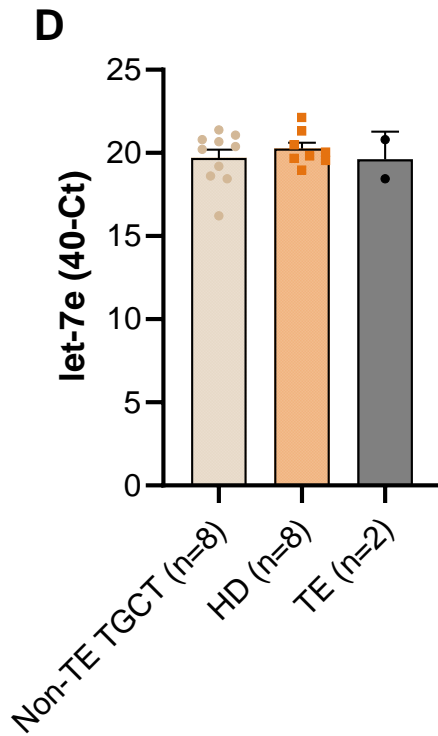

Supplement: Supplementary file 1 — Supplementary Material 1: Figure S1: Graphical representation of ATRA treatment schematic used for in the NT2 cell line. Figure S2: Total number of particles/ml (A, B), and mode particle size (C, D) for all separate (T)GCT cell lines lEV and sEV NTA experiments. Data shown as mean ± SEM for 3 independent experiments. Figure S3: RNA concentration measurement for the (T)GCT cell line-derived EV populations (A) and RNA concentration normalized to the number of particles: RNA per particle ratio (B). Data shown as mean ± SEM; * - p < 0.05. Figure S4: Raw figures of the western blots performed for the cell and tissue-derived EVs. Red arrow pinpoint blots that were represented in Figure 2A. Figure S5: Raw figures of the western blots performed for the plasma-derived EVs. Red arrow pinpoint blots that were represented in Figure 2B. Figure S6: Representative bright field microscopy imaging of NT2 cells 5 and 10 days after treatment start with vehicle (A,B) and ATRA (C,D). Figure S7: Western blot for pluripotency-related factors NANOG and PAX6, and for Beta-actin in vehicle and ATRA-treated NT2 cells. Figure S8: Raw figures of the western blots performed for the ATRA-treated cells. Red arrow pinpoint blots that were represented in Figure S7. Figure S9: Secretion range (NTA particles per cell ratio) for vehicle and ATRA-treated cells, in lEV (A) and sEV (B) populations. Data shown as mean ± SEM for 5 independent experiments; * - p < 0.05. Figure S10: RNA concentration measurements in tumor tissue vs non-tumoral adjacent tissue, in lEV (A) and sEV (B) populations. Data shown as mean ± SEM for 5 independent experiments; ** - p < 0.01. Figure S11: Tissue cellular levels for miR-371a-3p (A), miR-372-3p (B), miR-373-3p (C) and let-7e (D), in non-TE TGCT, TE and adjacent testicular parenchyma tissues. Data shown on a log scale as mean ± SEM; * - p < 0.05, *** -p < 0.001. Figure S12: Spearman correlation analysis for tissue cellular and conditioned medium (CM) lEV and sEV microRNA le [file 12964_2025_2250_MOESM1_ESM.zip › FigureS14.pdf]

**A**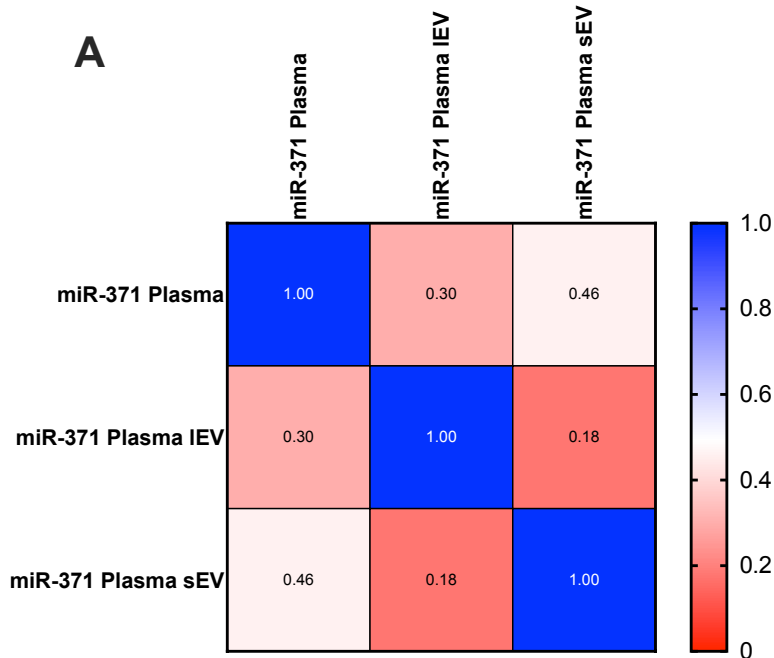**B**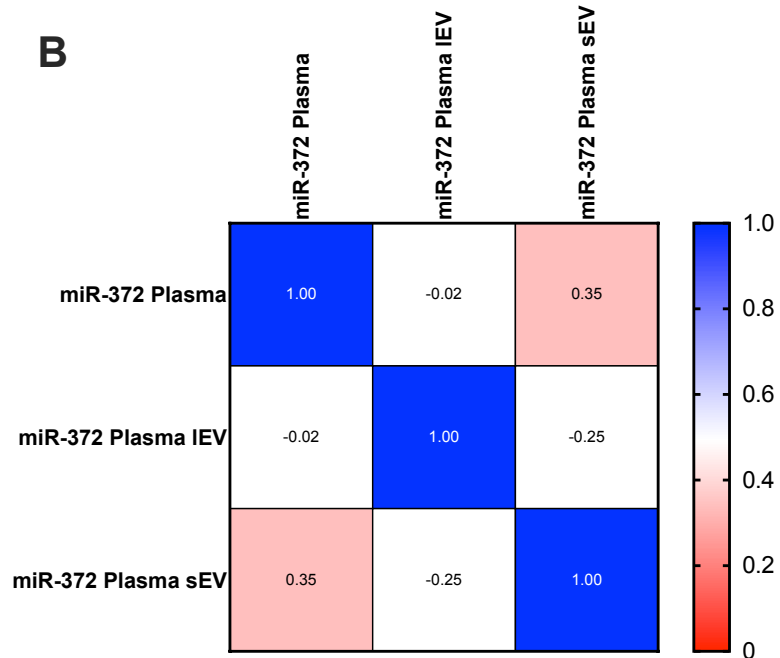**C**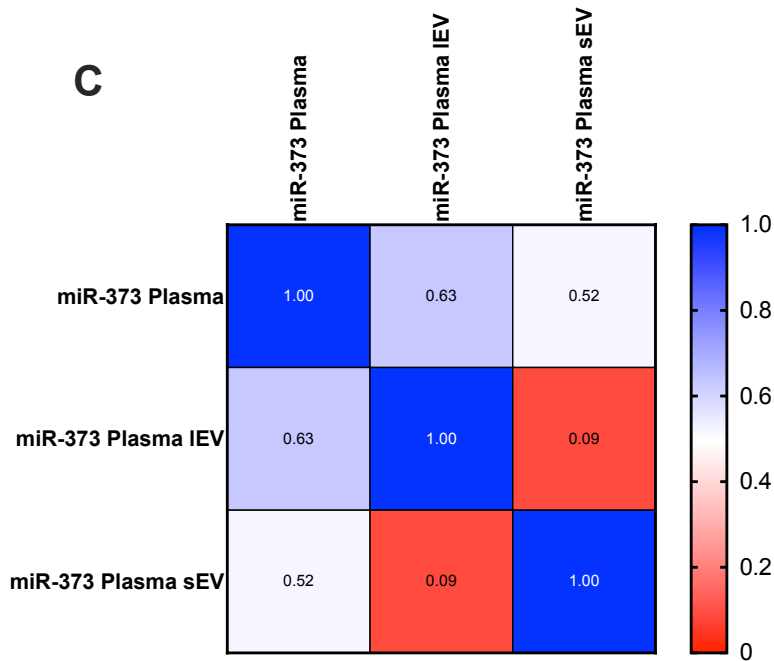**D**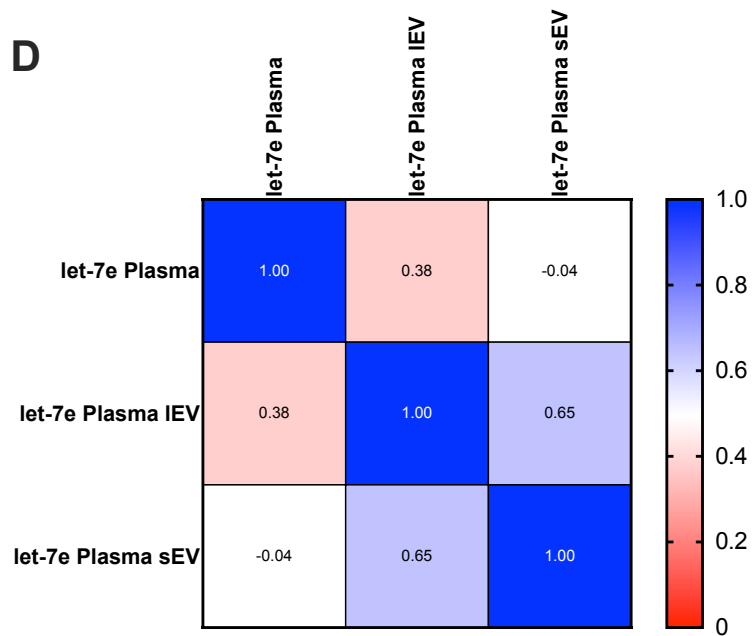

Supplement: Supplementary file 1 — Supplementary Material 1: Figure S1: Graphical representation of ATRA treatment schematic used for in the NT2 cell line. Figure S2: Total number of particles/ml (A, B), and mode particle size (C, D) for all separate (T)GCT cell lines lEV and sEV NTA experiments. Data shown as mean ± SEM for 3 independent experiments. Figure S3: RNA concentration measurement for the (T)GCT cell line-derived EV populations (A) and RNA concentration normalized to the number of particles: RNA per particle ratio (B). Data shown as mean ± SEM; * - p < 0.05. Figure S4: Raw figures of the western blots performed for the cell and tissue-derived EVs. Red arrow pinpoint blots that were represented in Figure 2A. Figure S5: Raw figures of the western blots performed for the plasma-derived EVs. Red arrow pinpoint blots that were represented in Figure 2B. Figure S6: Representative bright field microscopy imaging of NT2 cells 5 and 10 days after treatment start with vehicle (A,B) and ATRA (C,D). Figure S7: Western blot for pluripotency-related factors NANOG and PAX6, and for Beta-actin in vehicle and ATRA-treated NT2 cells. Figure S8: Raw figures of the western blots performed for the ATRA-treated cells. Red arrow pinpoint blots that were represented in Figure S7. Figure S9: Secretion range (NTA particles per cell ratio) for vehicle and ATRA-treated cells, in lEV (A) and sEV (B) populations. Data shown as mean ± SEM for 5 independent experiments; * - p < 0.05. Figure S10: RNA concentration measurements in tumor tissue vs non-tumoral adjacent tissue, in lEV (A) and sEV (B) populations. Data shown as mean ± SEM for 5 independent experiments; ** - p < 0.01. Figure S11: Tissue cellular levels for miR-371a-3p (A), miR-372-3p (B), miR-373-3p (C) and let-7e (D), in non-TE TGCT, TE and adjacent testicular parenchyma tissues. Data shown on a log scale as mean ± SEM; * - p < 0.05, *** -p < 0.001. Figure S12: Spearman correlation analysis for tissue cellular and conditioned medium (CM) lEV and sEV microRNA le [file 12964_2025_2250_MOESM1_ESM.zip › FigureS15.pdf]

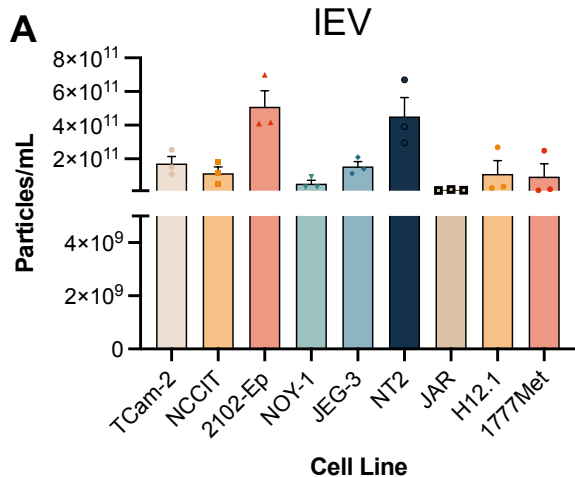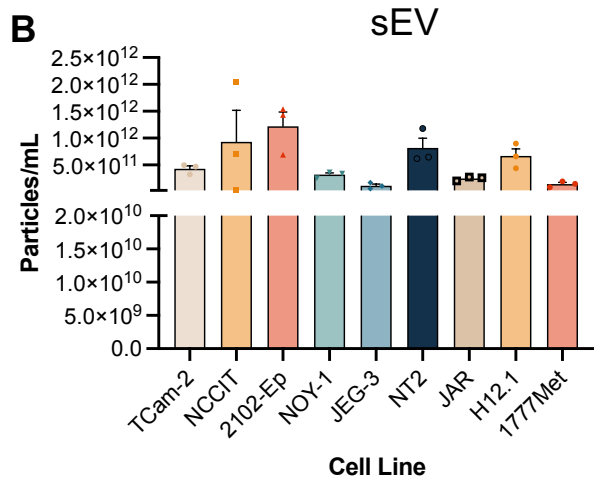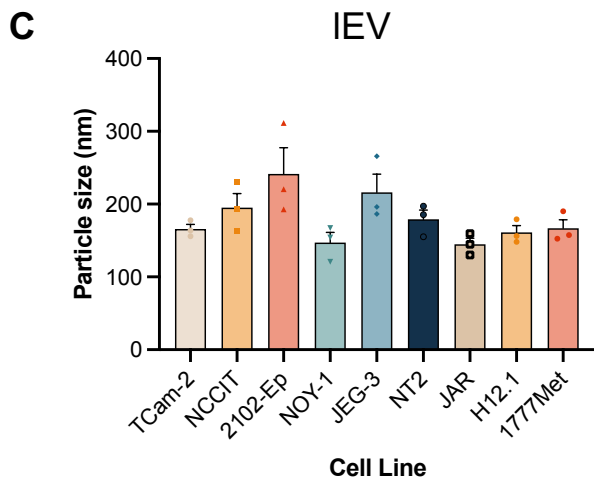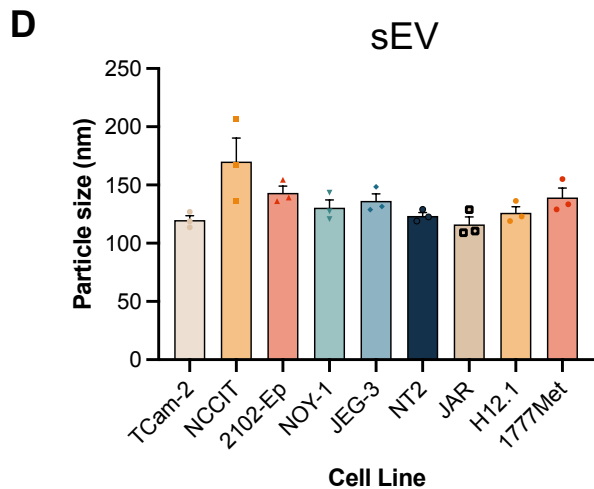

Supplement: Supplementary file 1 — Supplementary Material 1: Figure S1: Graphical representation of ATRA treatment schematic used for in the NT2 cell line. Figure S2: Total number of particles/ml (A, B), and mode particle size (C, D) for all separate (T)GCT cell lines lEV and sEV NTA experiments. Data shown as mean ± SEM for 3 independent experiments. Figure S3: RNA concentration measurement for the (T)GCT cell line-derived EV populations (A) and RNA concentration normalized to the number of particles: RNA per particle ratio (B). Data shown as mean ± SEM; * - p < 0.05. Figure S4: Raw figures of the western blots performed for the cell and tissue-derived EVs. Red arrow pinpoint blots that were represented in Figure 2A. Figure S5: Raw figures of the western blots performed for the plasma-derived EVs. Red arrow pinpoint blots that were represented in Figure 2B. Figure S6: Representative bright field microscopy imaging of NT2 cells 5 and 10 days after treatment start with vehicle (A,B) and ATRA (C,D). Figure S7: Western blot for pluripotency-related factors NANOG and PAX6, and for Beta-actin in vehicle and ATRA-treated NT2 cells. Figure S8: Raw figures of the western blots performed for the ATRA-treated cells. Red arrow pinpoint blots that were represented in Figure S7. Figure S9: Secretion range (NTA particles per cell ratio) for vehicle and ATRA-treated cells, in lEV (A) and sEV (B) populations. Data shown as mean ± SEM for 5 independent experiments; * - p < 0.05. Figure S10: RNA concentration measurements in tumor tissue vs non-tumoral adjacent tissue, in lEV (A) and sEV (B) populations. Data shown as mean ± SEM for 5 independent experiments; ** - p < 0.01. Figure S11: Tissue cellular levels for miR-371a-3p (A), miR-372-3p (B), miR-373-3p (C) and let-7e (D), in non-TE TGCT, TE and adjacent testicular parenchyma tissues. Data shown on a log scale as mean ± SEM; * - p < 0.05, *** -p < 0.001. Figure S12: Spearman correlation analysis for tissue cellular and conditioned medium (CM) lEV and sEV microRNA le [file 12964_2025_2250_MOESM1_ESM.zip › FigureS2.pdf]

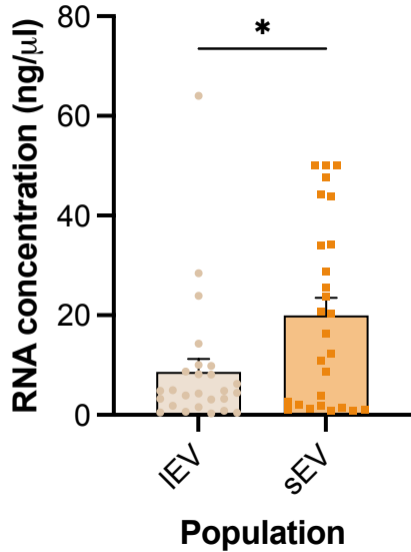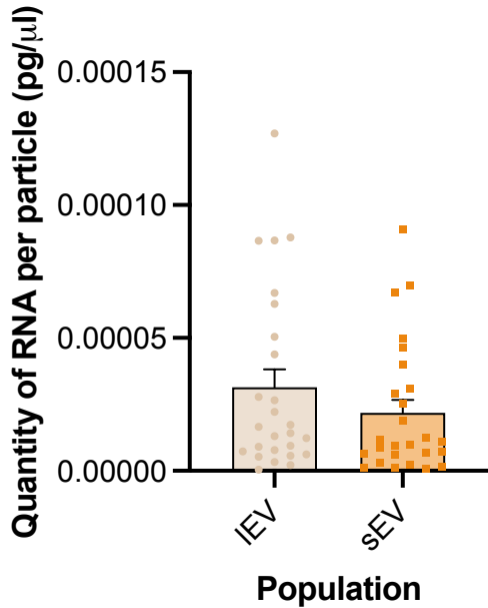

Supplement: Supplementary file 1 — Supplementary Material 1: Figure S1: Graphical representation of ATRA treatment schematic used for in the NT2 cell line. Figure S2: Total number of particles/ml (A, B), and mode particle size (C, D) for all separate (T)GCT cell lines lEV and sEV NTA experiments. Data shown as mean ± SEM for 3 independent experiments. Figure S3: RNA concentration measurement for the (T)GCT cell line-derived EV populations (A) and RNA concentration normalized to the number of particles: RNA per particle ratio (B). Data shown as mean ± SEM; * - p < 0.05. Figure S4: Raw figures of the western blots performed for the cell and tissue-derived EVs. Red arrow pinpoint blots that were represented in Figure 2A. Figure S5: Raw figures of the western blots performed for the plasma-derived EVs. Red arrow pinpoint blots that were represented in Figure 2B. Figure S6: Representative bright field microscopy imaging of NT2 cells 5 and 10 days after treatment start with vehicle (A,B) and ATRA (C,D). Figure S7: Western blot for pluripotency-related factors NANOG and PAX6, and for Beta-actin in vehicle and ATRA-treated NT2 cells. Figure S8: Raw figures of the western blots performed for the ATRA-treated cells. Red arrow pinpoint blots that were represented in Figure S7. Figure S9: Secretion range (NTA particles per cell ratio) for vehicle and ATRA-treated cells, in lEV (A) and sEV (B) populations. Data shown as mean ± SEM for 5 independent experiments; * - p < 0.05. Figure S10: RNA concentration measurements in tumor tissue vs non-tumoral adjacent tissue, in lEV (A) and sEV (B) populations. Data shown as mean ± SEM for 5 independent experiments; ** - p < 0.01. Figure S11: Tissue cellular levels for miR-371a-3p (A), miR-372-3p (B), miR-373-3p (C) and let-7e (D), in non-TE TGCT, TE and adjacent testicular parenchyma tissues. Data shown on a log scale as mean ± SEM; * - p < 0.05, *** -p < 0.001. Figure S12: Spearman correlation analysis for tissue cellular and conditioned medium (CM) lEV and sEV microRNA le [file 12964_2025_2250_MOESM1_ESM.zip › FigureS3.pdf]

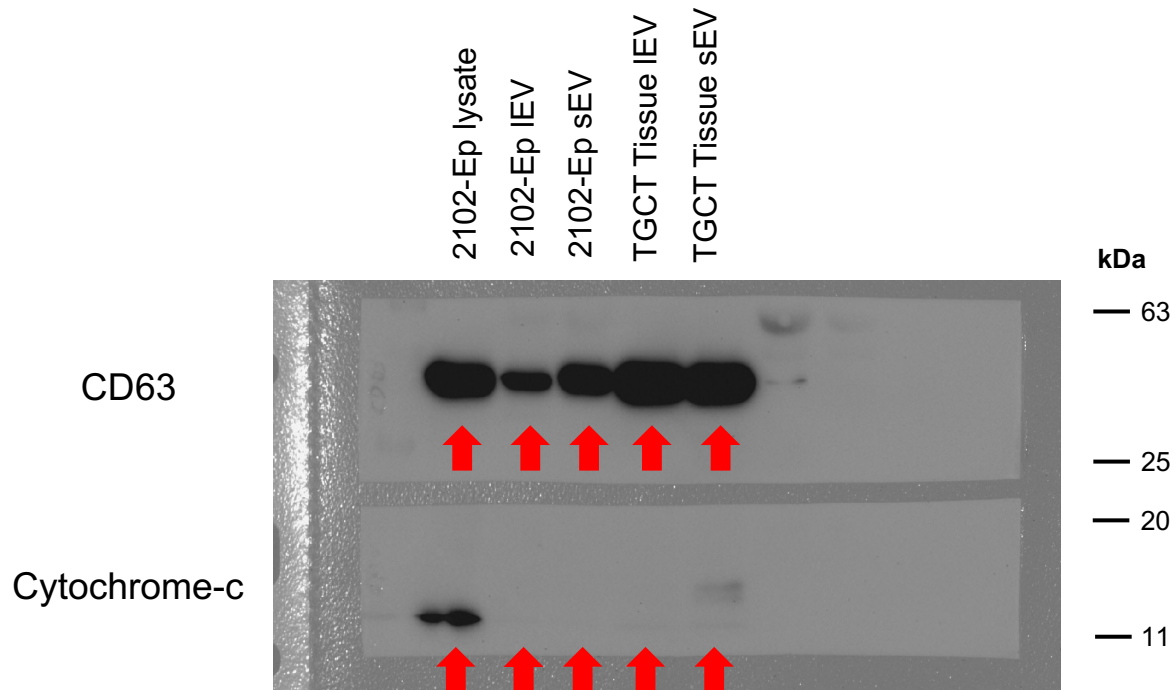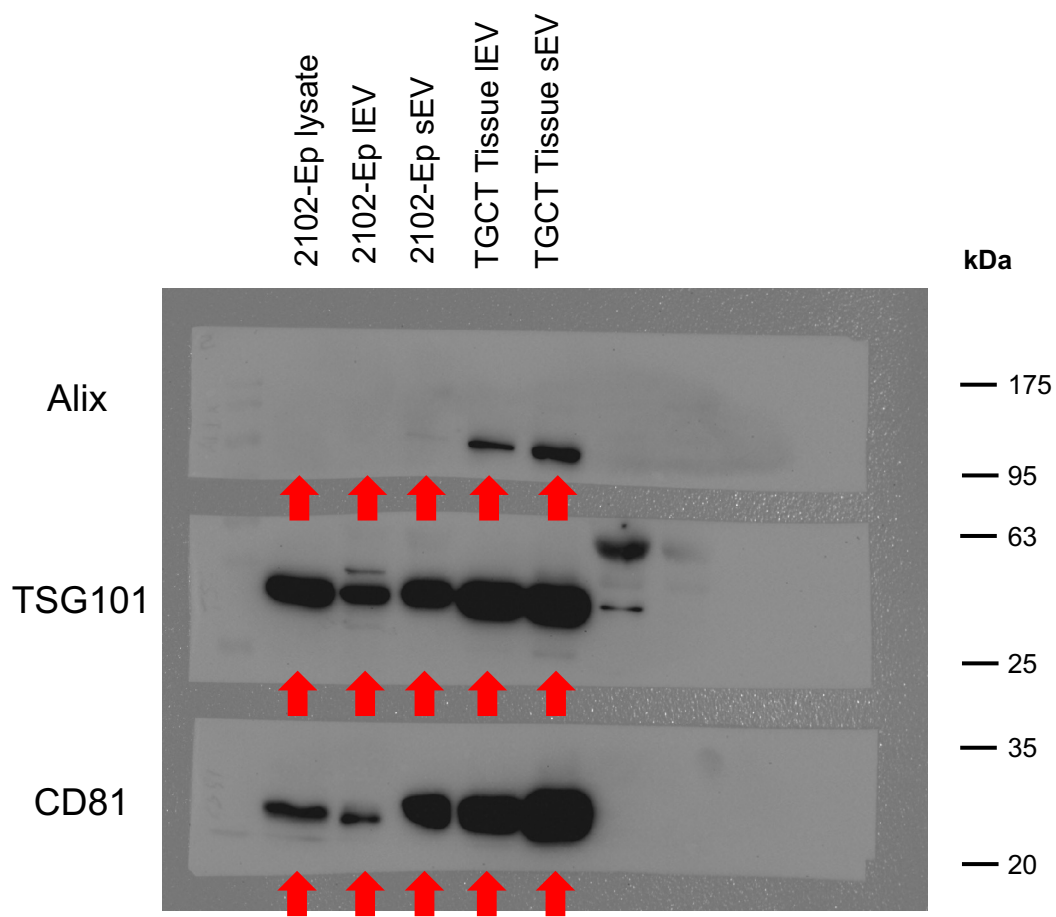

Supplement: Supplementary file 1 — Supplementary Material 1: Figure S1: Graphical representation of ATRA treatment schematic used for in the NT2 cell line. Figure S2: Total number of particles/ml (A, B), and mode particle size (C, D) for all separate (T)GCT cell lines lEV and sEV NTA experiments. Data shown as mean ± SEM for 3 independent experiments. Figure S3: RNA concentration measurement for the (T)GCT cell line-derived EV populations (A) and RNA concentration normalized to the number of particles: RNA per particle ratio (B). Data shown as mean ± SEM; * - p < 0.05. Figure S4: Raw figures of the western blots performed for the cell and tissue-derived EVs. Red arrow pinpoint blots that were represented in Figure 2A. Figure S5: Raw figures of the western blots performed for the plasma-derived EVs. Red arrow pinpoint blots that were represented in Figure 2B. Figure S6: Representative bright field microscopy imaging of NT2 cells 5 and 10 days after treatment start with vehicle (A,B) and ATRA (C,D). Figure S7: Western blot for pluripotency-related factors NANOG and PAX6, and for Beta-actin in vehicle and ATRA-treated NT2 cells. Figure S8: Raw figures of the western blots performed for the ATRA-treated cells. Red arrow pinpoint blots that were represented in Figure S7. Figure S9: Secretion range (NTA particles per cell ratio) for vehicle and ATRA-treated cells, in lEV (A) and sEV (B) populations. Data shown as mean ± SEM for 5 independent experiments; * - p < 0.05. Figure S10: RNA concentration measurements in tumor tissue vs non-tumoral adjacent tissue, in lEV (A) and sEV (B) populations. Data shown as mean ± SEM for 5 independent experiments; ** - p < 0.01. Figure S11: Tissue cellular levels for miR-371a-3p (A), miR-372-3p (B), miR-373-3p (C) and let-7e (D), in non-TE TGCT, TE and adjacent testicular parenchyma tissues. Data shown on a log scale as mean ± SEM; * - p < 0.05, *** -p < 0.001. Figure S12: Spearman correlation analysis for tissue cellular and conditioned medium (CM) lEV and sEV microRNA le [file 12964_2025_2250_MOESM1_ESM.zip › FigureS4.pdf]

2102-Ep lysate  
TGCT plasma IEV  
TGCT plasma sEV

kDa

Albumin

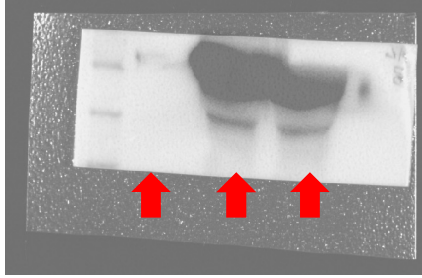

— 63

— 35

HSC70

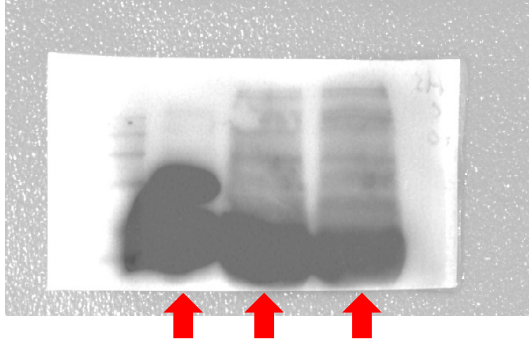

— 100

— 63

CD9

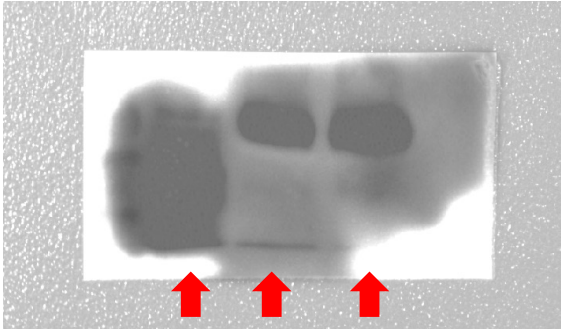

— 35

— 17

CD81

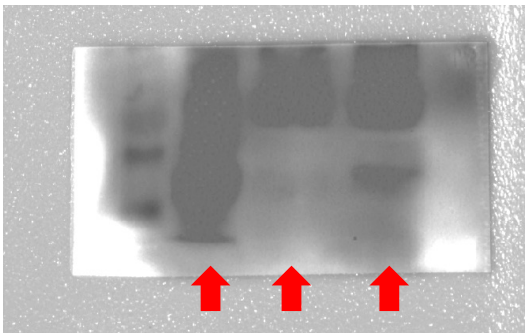

— 35

— 17

Supplement: Supplementary file 1 — Supplementary Material 1: Figure S1: Graphical representation of ATRA treatment schematic used for in the NT2 cell line. Figure S2: Total number of particles/ml (A, B), and mode particle size (C, D) for all separate (T)GCT cell lines lEV and sEV NTA experiments. Data shown as mean ± SEM for 3 independent experiments. Figure S3: RNA concentration measurement for the (T)GCT cell line-derived EV populations (A) and RNA concentration normalized to the number of particles: RNA per particle ratio (B). Data shown as mean ± SEM; * - p < 0.05. Figure S4: Raw figures of the western blots performed for the cell and tissue-derived EVs. Red arrow pinpoint blots that were represented in Figure 2A. Figure S5: Raw figures of the western blots performed for the plasma-derived EVs. Red arrow pinpoint blots that were represented in Figure 2B. Figure S6: Representative bright field microscopy imaging of NT2 cells 5 and 10 days after treatment start with vehicle (A,B) and ATRA (C,D). Figure S7: Western blot for pluripotency-related factors NANOG and PAX6, and for Beta-actin in vehicle and ATRA-treated NT2 cells. Figure S8: Raw figures of the western blots performed for the ATRA-treated cells. Red arrow pinpoint blots that were represented in Figure S7. Figure S9: Secretion range (NTA particles per cell ratio) for vehicle and ATRA-treated cells, in lEV (A) and sEV (B) populations. Data shown as mean ± SEM for 5 independent experiments; * - p < 0.05. Figure S10: RNA concentration measurements in tumor tissue vs non-tumoral adjacent tissue, in lEV (A) and sEV (B) populations. Data shown as mean ± SEM for 5 independent experiments; ** - p < 0.01. Figure S11: Tissue cellular levels for miR-371a-3p (A), miR-372-3p (B), miR-373-3p (C) and let-7e (D), in non-TE TGCT, TE and adjacent testicular parenchyma tissues. Data shown on a log scale as mean ± SEM; * - p < 0.05, *** -p < 0.001. Figure S12: Spearman correlation analysis for tissue cellular and conditioned medium (CM) lEV and sEV microRNA le [file 12964_2025_2250_MOESM1_ESM.zip › FigureS5.pdf]

5 days

10 days

Vehicle

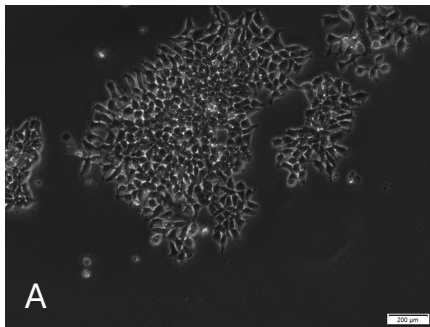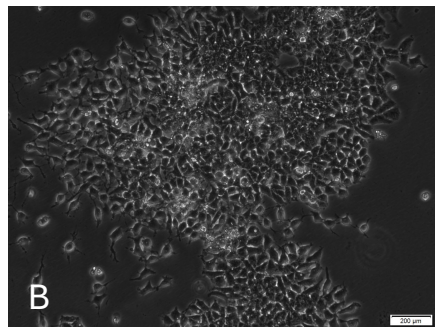

ATRA

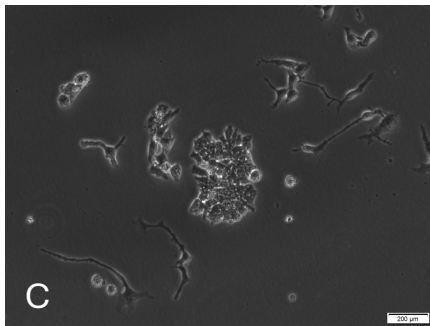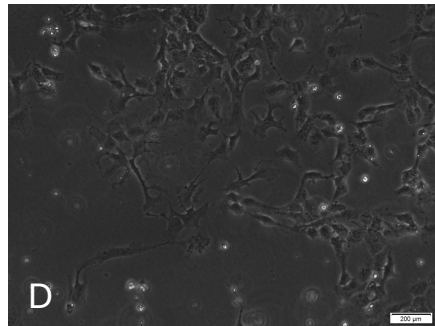

Supplement: Supplementary file 1 — Supplementary Material 1: Figure S1: Graphical representation of ATRA treatment schematic used for in the NT2 cell line. Figure S2: Total number of particles/ml (A, B), and mode particle size (C, D) for all separate (T)GCT cell lines lEV and sEV NTA experiments. Data shown as mean ± SEM for 3 independent experiments. Figure S3: RNA concentration measurement for the (T)GCT cell line-derived EV populations (A) and RNA concentration normalized to the number of particles: RNA per particle ratio (B). Data shown as mean ± SEM; * - p < 0.05. Figure S4: Raw figures of the western blots performed for the cell and tissue-derived EVs. Red arrow pinpoint blots that were represented in Figure 2A. Figure S5: Raw figures of the western blots performed for the plasma-derived EVs. Red arrow pinpoint blots that were represented in Figure 2B. Figure S6: Representative bright field microscopy imaging of NT2 cells 5 and 10 days after treatment start with vehicle (A,B) and ATRA (C,D). Figure S7: Western blot for pluripotency-related factors NANOG and PAX6, and for Beta-actin in vehicle and ATRA-treated NT2 cells. Figure S8: Raw figures of the western blots performed for the ATRA-treated cells. Red arrow pinpoint blots that were represented in Figure S7. Figure S9: Secretion range (NTA particles per cell ratio) for vehicle and ATRA-treated cells, in lEV (A) and sEV (B) populations. Data shown as mean ± SEM for 5 independent experiments; * - p < 0.05. Figure S10: RNA concentration measurements in tumor tissue vs non-tumoral adjacent tissue, in lEV (A) and sEV (B) populations. Data shown as mean ± SEM for 5 independent experiments; ** - p < 0.01. Figure S11: Tissue cellular levels for miR-371a-3p (A), miR-372-3p (B), miR-373-3p (C) and let-7e (D), in non-TE TGCT, TE and adjacent testicular parenchyma tissues. Data shown on a log scale as mean ± SEM; * - p < 0.05, *** -p < 0.001. Figure S12: Spearman correlation analysis for tissue cellular and conditioned medium (CM) lEV and sEV microRNA le [file 12964_2025_2250_MOESM1_ESM.zip › FigureS6.pdf]

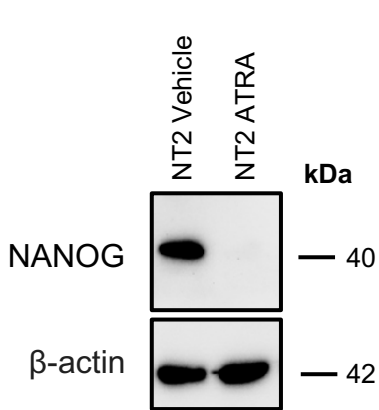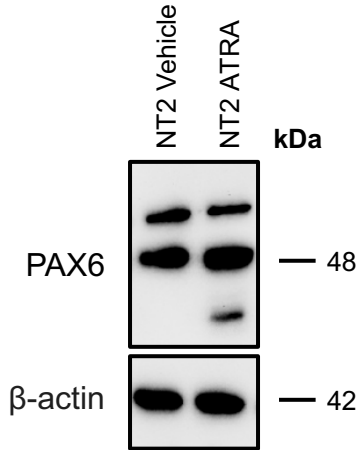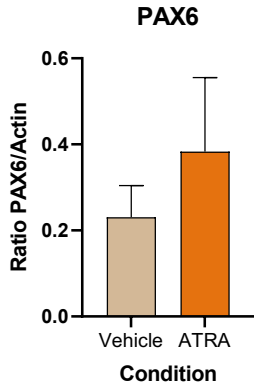

Supplement: Supplementary file 1 — Supplementary Material 1: Figure S1: Graphical representation of ATRA treatment schematic used for in the NT2 cell line. Figure S2: Total number of particles/ml (A, B), and mode particle size (C, D) for all separate (T)GCT cell lines lEV and sEV NTA experiments. Data shown as mean ± SEM for 3 independent experiments. Figure S3: RNA concentration measurement for the (T)GCT cell line-derived EV populations (A) and RNA concentration normalized to the number of particles: RNA per particle ratio (B). Data shown as mean ± SEM; * - p < 0.05. Figure S4: Raw figures of the western blots performed for the cell and tissue-derived EVs. Red arrow pinpoint blots that were represented in Figure 2A. Figure S5: Raw figures of the western blots performed for the plasma-derived EVs. Red arrow pinpoint blots that were represented in Figure 2B. Figure S6: Representative bright field microscopy imaging of NT2 cells 5 and 10 days after treatment start with vehicle (A,B) and ATRA (C,D). Figure S7: Western blot for pluripotency-related factors NANOG and PAX6, and for Beta-actin in vehicle and ATRA-treated NT2 cells. Figure S8: Raw figures of the western blots performed for the ATRA-treated cells. Red arrow pinpoint blots that were represented in Figure S7. Figure S9: Secretion range (NTA particles per cell ratio) for vehicle and ATRA-treated cells, in lEV (A) and sEV (B) populations. Data shown as mean ± SEM for 5 independent experiments; * - p < 0.05. Figure S10: RNA concentration measurements in tumor tissue vs non-tumoral adjacent tissue, in lEV (A) and sEV (B) populations. Data shown as mean ± SEM for 5 independent experiments; ** - p < 0.01. Figure S11: Tissue cellular levels for miR-371a-3p (A), miR-372-3p (B), miR-373-3p (C) and let-7e (D), in non-TE TGCT, TE and adjacent testicular parenchyma tissues. Data shown on a log scale as mean ± SEM; * - p < 0.05, *** -p < 0.001. Figure S12: Spearman correlation analysis for tissue cellular and conditioned medium (CM) lEV and sEV microRNA le [file 12964_2025_2250_MOESM1_ESM.zip › FigureS7.pdf]

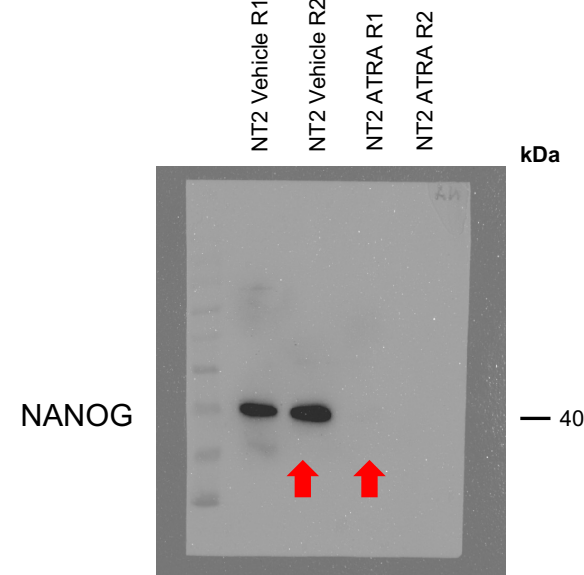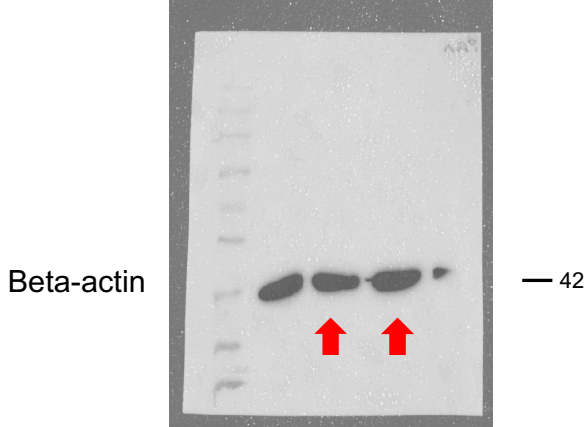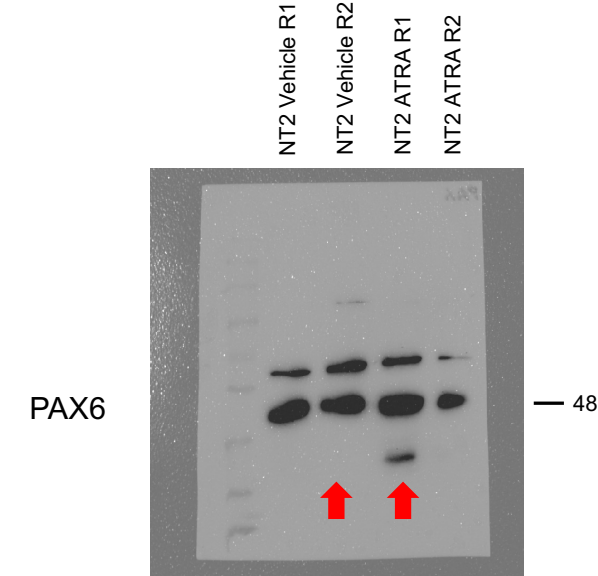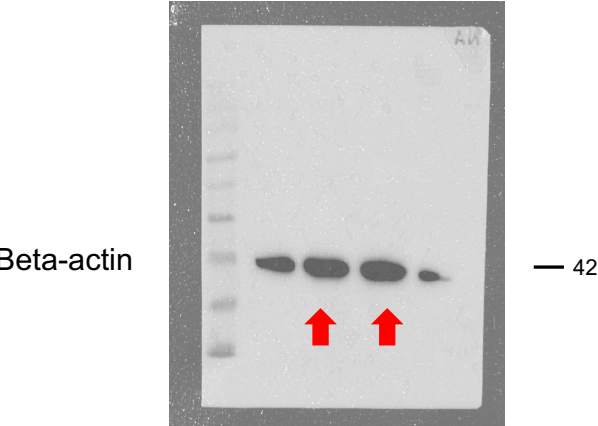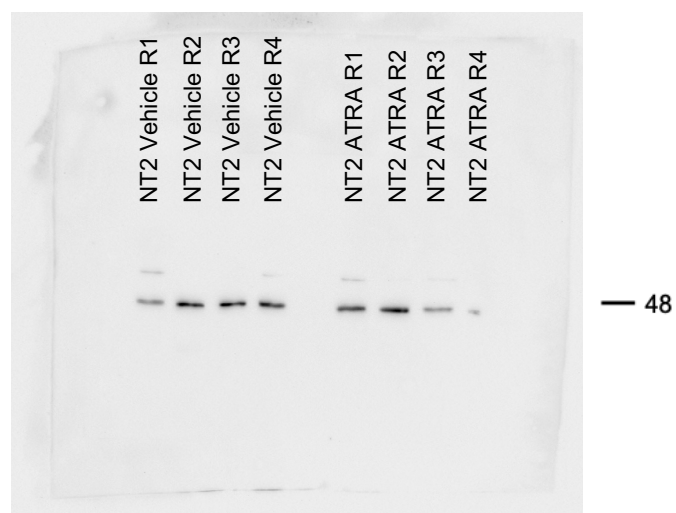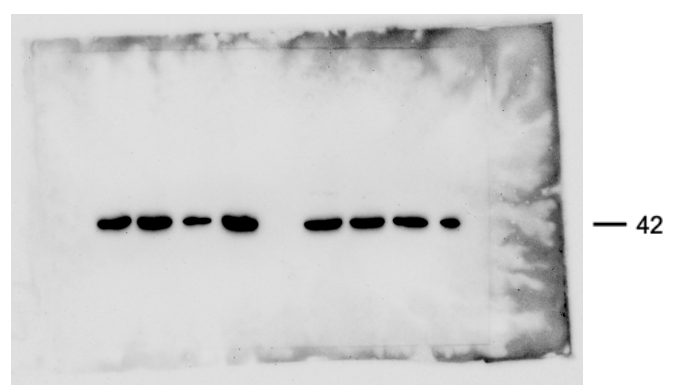

Supplement: Supplementary file 1 — Supplementary Material 1: Figure S1: Graphical representation of ATRA treatment schematic used for in the NT2 cell line. Figure S2: Total number of particles/ml (A, B), and mode particle size (C, D) for all separate (T)GCT cell lines lEV and sEV NTA experiments. Data shown as mean ± SEM for 3 independent experiments. Figure S3: RNA concentration measurement for the (T)GCT cell line-derived EV populations (A) and RNA concentration normalized to the number of particles: RNA per particle ratio (B). Data shown as mean ± SEM; * - p < 0.05. Figure S4: Raw figures of the western blots performed for the cell and tissue-derived EVs. Red arrow pinpoint blots that were represented in Figure 2A. Figure S5: Raw figures of the western blots performed for the plasma-derived EVs. Red arrow pinpoint blots that were represented in Figure 2B. Figure S6: Representative bright field microscopy imaging of NT2 cells 5 and 10 days after treatment start with vehicle (A,B) and ATRA (C,D). Figure S7: Western blot for pluripotency-related factors NANOG and PAX6, and for Beta-actin in vehicle and ATRA-treated NT2 cells. Figure S8: Raw figures of the western blots performed for the ATRA-treated cells. Red arrow pinpoint blots that were represented in Figure S7. Figure S9: Secretion range (NTA particles per cell ratio) for vehicle and ATRA-treated cells, in lEV (A) and sEV (B) populations. Data shown as mean ± SEM for 5 independent experiments; * - p < 0.05. Figure S10: RNA concentration measurements in tumor tissue vs non-tumoral adjacent tissue, in lEV (A) and sEV (B) populations. Data shown as mean ± SEM for 5 independent experiments; ** - p < 0.01. Figure S11: Tissue cellular levels for miR-371a-3p (A), miR-372-3p (B), miR-373-3p (C) and let-7e (D), in non-TE TGCT, TE and adjacent testicular parenchyma tissues. Data shown on a log scale as mean ± SEM; * - p < 0.05, *** -p < 0.001. Figure S12: Spearman correlation analysis for tissue cellular and conditioned medium (CM) lEV and sEV microRNA le [file 12964_2025_2250_MOESM1_ESM.zip › FigureS8.pdf]

IEV

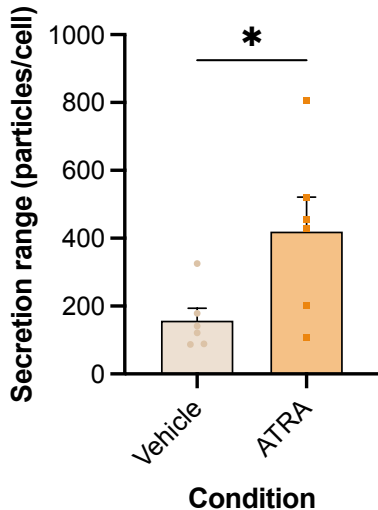

sEV

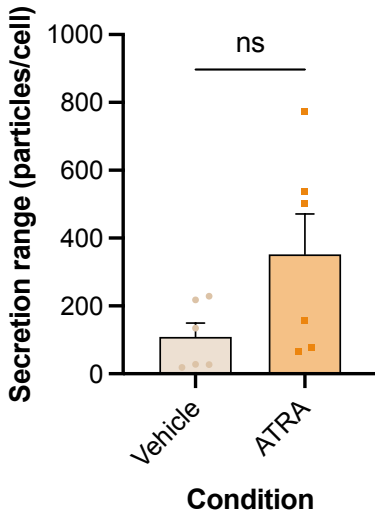

Supplement: Supplementary file 1 — Supplementary Material 1: Figure S1: Graphical representation of ATRA treatment schematic used for in the NT2 cell line. Figure S2: Total number of particles/ml (A, B), and mode particle size (C, D) for all separate (T)GCT cell lines lEV and sEV NTA experiments. Data shown as mean ± SEM for 3 independent experiments. Figure S3: RNA concentration measurement for the (T)GCT cell line-derived EV populations (A) and RNA concentration normalized to the number of particles: RNA per particle ratio (B). Data shown as mean ± SEM; * - p < 0.05. Figure S4: Raw figures of the western blots performed for the cell and tissue-derived EVs. Red arrow pinpoint blots that were represented in Figure 2A. Figure S5: Raw figures of the western blots performed for the plasma-derived EVs. Red arrow pinpoint blots that were represented in Figure 2B. Figure S6: Representative bright field microscopy imaging of NT2 cells 5 and 10 days after treatment start with vehicle (A,B) and ATRA (C,D). Figure S7: Western blot for pluripotency-related factors NANOG and PAX6, and for Beta-actin in vehicle and ATRA-treated NT2 cells. Figure S8: Raw figures of the western blots performed for the ATRA-treated cells. Red arrow pinpoint blots that were represented in Figure S7. Figure S9: Secretion range (NTA particles per cell ratio) for vehicle and ATRA-treated cells, in lEV (A) and sEV (B) populations. Data shown as mean ± SEM for 5 independent experiments; * - p < 0.05. Figure S10: RNA concentration measurements in tumor tissue vs non-tumoral adjacent tissue, in lEV (A) and sEV (B) populations. Data shown as mean ± SEM for 5 independent experiments; ** - p < 0.01. Figure S11: Tissue cellular levels for miR-371a-3p (A), miR-372-3p (B), miR-373-3p (C) and let-7e (D), in non-TE TGCT, TE and adjacent testicular parenchyma tissues. Data shown on a log scale as mean ± SEM; * - p < 0.05, *** -p < 0.001. Figure S12: Spearman correlation analysis for tissue cellular and conditioned medium (CM) lEV and sEV microRNA le [file 12964_2025_2250_MOESM1_ESM.zip › FigureS9.pdf]
