# Supplementary material for: MicroRNA-371–373 cluster extracellular vesicle-based communication in testicular germ cell tumors
Source: Cell Commun Signal. 2025 May 30;23:252. doi: 10.1186/s12964-025-02250-8 (PMC12124061; doi:10.1186/s12964-025-02250-8)
Supplement: Supplementary file 2 — Supplementary Material 2: Table 1: (T)GCT cell lines used in the study. Table 2: Clinical and histopathological parameters of the patients included in the study. Arrows (⇑) represent abnormal elevation of the classical serum tumor markers. Asterisks (*) represent the histological components assessed in the evaluated tissue specimens for mixed NS GCTs. Table 3: List of primers used for RT-qPCR experiments. Table 4: List of antibodies used for the western blot experiments. [file 12964_2025_2250_MOESM2_ESM.docx]

**MicroRNA-371-373 cluster extracellular vesicle-based communication in testicular germ cell tumors**

Nuno Tiago Tavares^1,2^, Catarina Lourenço^1,2,3^, Vera Constâncio^1,2^, Fernanda Fernandes-Pontes^1^, Diana Fonseca^1^, Rui Silva-Santos^4^, Isaac Braga^5^, Joaquina Maurício^6^, Rui Henrique^1,4,7^, Michelle Liu^8^, Robert S. Weiss^8^, Aditya Bagrodia^9^, Carmen Jerónimo^1,7*^, João Lobo^1,4,7*#^

1 - Cancer Biology and Epigenetics Group, Research Center of IPO Porto CI-IPOP/CI-IPOP@RISE Health Research Network- Portuguese Oncology Institute of Porto IPO Porto/Porto Comprehensive Cancer Center Raquel Seruca Porto.CCC Raquel Seruca- 4200-072 Porto- Portugal.

2 - Doctoral Programme in Biomedical Sciences, School of Medicine and Biomedical Sciences- University of Porto (ICBAS-UP), 4050-313 Porto- Portugal.

3 - i3S-Instituto de Investigação e Inovação em Saúde- Universidade do Porto- 4200-135 Porto- Portugal, Instituto Nacional de Engenharia Biomédica, Portugal, Portugal.

4 - Department of Pathology, Portuguese Oncology Institute of Porto (IPO Porto) 4200-072 Porto- Portugal.

5 – Urology Clinic, Department of Medical Oncology, Portuguese Oncology Institute of Porto IPO Porto- 4200-072 Porto- Portugal.

6 – Urology Clinic, Department of Urology, Portuguese Oncology Institute of Porto IPO Porto- 4200-072 Porto- Portugal.

7 - Department of Pathology and Molecular Immunology, School of Medicine & Biomedical Sciences - University of Porto (ICBAS-UP), 4050-313 Porto- Portugal.

8 - Department of Biomedical Sciences, College of Veterinary Medicine, Cornell University, Ithaca, NY, USA.

9 - Department of Urology, University of California San Diego, San Diego – CA – USA.

* - co-senior authors.

# - Corresponding author: João Lobo, MD, PhD. Department of Pathology, Portuguese Oncology Institute of Porto (IPO Porto), Rua Dr. António Bernardino de Almeida, 4200-072 Porto, Portugal

E-mail: [joaomachadolobo@gmail.com](mailto:joaomachadolobo@gmail.com) / [jpedro.lobo@ipoporto.min-saude.pt](mailto:jpedro.lobo@ipoporto.min-saude.pt)

**Abstract**

Testicular germ cell tumors (TGCTs) represent the most common type of cancer in young adults. The cluster of microRNAs 371-373 is highly upregulated in TGCTs, and detection of miR-371a-3p specifically is currently being developed for clinical implementation as a sensitive and specific biomarker for TGCT, except for teratoma. Extracellular vesicles (EVs) are nano-sized particles used for cell communication, being increasingly regarded as potential sources of cancer biomarkers. Thus, the aim of this study was to characterize EVs from a wide range of TGCT samples, including cell lines, tissue explants and matched plasma samples from patients and healthy donors, and then use these samples to assess microRNA expression (miR-371-373 cluster and let-7e). TGCT-derived EVs were successfully isolated and characterized according to MISEV guidelines. TGCT cell lines showed different levels of EV-derived miR-371-373 cluster and let-7e. Upon differentiation of NT2 cells with ATRA, both cellular and EV-derived miR-371-373 cluster were downregulated, whereas let-7e was upregulated. TGCT patient samples presented high levels of EV-derived miR-371-373, except for the teratoma samples. We conclude that a significant portion of the circulating miR-371-373 cluster used as a TGCT biomarker in the clinic is secreted into EVs, and that this cluster and the let-7 family of microRNAs may be related with TGCT intercellular communication and differentiation.

**Introduction**

Testicular germ cell tumors (TGCTs) are the most common type of malignancies in young adult males, with increasing incidence worldwide in the last years, in part due to lifestyle factors [1, 2]. These tumors are complex and heterogeneous, showing a background related to epigenetics and reflecting steps of germ cell development. TGCTs are divided into seminomas (SE) and non-seminomas (NS), with different follow-up and treatment algorithms [3]. NS include embryonal carcinoma (EC), yolk sac tumor (YST), choriocarcinoma (CHC), and teratoma (TE), as well as mixed tumors [4].

MicroRNAs (miRNAs) are small RNAs that are commonly dysregulated in several diseases, including in cancer [5, 6]. They have been studied in the last decades as cancer biomarkers, as they can be detected in tissue samples and body fluids, circulating with very high stability [7]. The miR-371-373 cluster is characterized as embryonic stem and germ cell specific, being highly upregulated in TGCTs [8, 9]. Specifically, circulating miR-371a-3p is highly sensitive and specific as a TGCT biomarker [10], except for TE histology, overcoming current limitations of the classical serum tumor markers (STMs). Its potential has been demonstrated in several studies with multicentric retrospective and prospective patient cohorts [11-19], leading to its approval as an *in vitro* diagnostic (IVD) test for use in the clinic. On the other hand, let-7 family miRNAs are tumor suppressor miRNAs that are known to be downregulated in TGCTs in conjunction with upregulation of Lin28, which encodes a negative regulator of let-7 miRNA processing and itself can be used as diagnostic marker for TGCTs [20, 21]. However, the secretion dynamics of these miRNAs in extracellular vesicles (EVs) derived from TGCT patients are still barely known.

EVs are small lipid bound particles secreted by cells into the extracellular space for intercellular communication [22]. They can be divided into apoptotic bodies, microvesicles and exosomes, according to their biogenesis, secretion pathways, function, cargo and size [22]. Their cargo includes proteins and various nucleic acids, such as DNA and different types of RNAs, including miRNAs [23, 24]. EV-derived miRNAs are highly protected from degradation, adding to their potential as robust and stable cancer biomarkers [25]. To date, only two studies report EV isolation from TGCTs, both from TGCT cell lines [26, 27], and no studies isolated EVs from representative clinical samples yet. Given the clinical use of miR-371a-3p as a cancer biomarker, studies characterizing TGCT-derived EVs from patient samples and assessing miR-371-373 cluster secretion processes are warranted.

In this work we successfully isolated and characterized EVs released from a wide range of TGCT samples, including cell lines, conditioned media from tissue explants (both tumor and adjacent testicular parenchyma) and matched plasma samples from the same patients, as well as age-matched male healthy blood donors. We then assessed microRNA expression (miR-371-373 cluster and let-7e), aiming to determine if these miRNAs are secreted in EVs derived from TGCT patient samples, and to verify if levels are concordant between matched tissue, blood sample sets and their respective EV-secreted miRNAs.

**Methods**

- 1. **Cell culture**

Nine (T)GCT cell lines were used for this study, representative of various histological types and disease stages. Characterization of the cell lines and corresponding used culture medium is provided in Supplementary Table 1. In order to perform EV isolation, cell lines were cultured in 5 150 mm Petri dishes with 20 ml of medium each (100 ml in total), supplemented with EV-free fetal bovine serum (FBS). FBS was purged from vesicles via ultracentrifugation at 100.000g for 1h10min. Cells were subsequently left to grow for 72 hours, to reach 80 to 90% confluence. Medium was then harvested, centrifuged twice to eliminate cellular debris, and stored at -80ºC, until EV isolation.

- 1. **Clinical samples**

A total of 14 pairs of patient samples (plasma and tissue samples) were prospectively collected and included in this study, along with 10 healthy donor plasma samples. Fresh tissue specimens from TGCT patients (n=6 SE and n=8 NS) were collected by a TGCT-dedicated pathologist at the operating room immediately after surgery. All patients were diagnosed and treated at the Portuguese Oncology Institute of Porto (IPO Porto) by the same multidisciplinary team, between 2022 and 2024. Surgical specimens consisted of radical inguinal orchiectomy (n=13) and/or retroperitoneal lymph node dissection (RPLND) (n=1). Samples were collected after obtaining patient informed consent (CES176/022). Clinical and histopathological characteristics of the patients included in the study are depicted in Supplementary Table 2. Paired plasma samples from TGCT patients were drawn right before orchiectomy or RPLND, in the same timepoint the tissue specimen was collected (i.e. all tissue and liquid biopsy samples in the study were matched, collected at the same time point, allowing for comparison between levels in tumor tissue and in circulation in each patient). All blood sample collections coincided with routine determinations of classical STMs (alpha fetoprotein [AFP], human chorionic gonadotropin [β-HCG] and lactate dehydrogenase [LDH]), which were available and compared to hsa-miR-371a-3p measurements. All healthy donors included in the study were males, age-matched with the patient group.

- 1. **Sample processing**

Tissue explant samples were cultured in 6 mL medium overnight, with an incubation time of a minimum 10 hours and a maximum 18 hours. Afterwards, tissues were flash-frozen and stored at -80ºC, and medium was harvested, centrifuged twice to eliminate tissue and cell debris, and stored at -80ºC for subsequent EV isolation.

Peripheral blood was collected in EDTA-containing tubes, and plasma was separated after centrifuging for 30 min at 2500 g (4ºC) and subsequently stored at -80ºC in the IPO Porto Department of Pathology Biobank. All blood samples were processed within a maximum of 4 hours after collection.

Clinical and histopathological data were reviewed by a TGCT-dedicated pathologist and according to the most recent WHO 2022 classification and AJCC 8^th^ edition staging manual [28]. All tissue specimens were histologically assessed using an H&E slide from the tissue piece that was in culture previously, that was verified by a TGCT-dedicated Pathologist (senior author, JL). Additionally, for better morphology assessment, a twin FFPE fragment was collected, to further perform histological characterization of the tumor piece used.

- 1. **Extracellular vesicles isolation and characterization**

6 mL of tissue culture media, 100 mL of cell culture media and 2 mL of plasma were used to perform EV isolation (volumes defined after optimization), using differential ultracentrifugation (UC), as previously described by [29]. Two fractions of EVs were isolated, one from the first UC pellet (12.000 g for 20 min), designated “large EVs (lEVs)” henceforth, and another from the second UC pellet (100.000g for 1h10min), designated “small EVs (sEVs)” hereafter. The two EV populations were characterized according to the Minimal Information for Studies of Extracellular Vesicles 2023 (MISEV2023) guidelines [30], using a combination of western blot, Nanoparticle Tracking Analysis (NTA) and transmission electron microscopy (TEM).

- - 1. **Protein quantification and western blot**

Protein concentration of cellular and EV samples was quantified using the BCA™ Protein Assay Kit (ThermoFisher Scientific, Waltham, Massachusetts, USA), following manufacturer’s instructions. The plates were read at 562 nm in a FLUOstar Omega microplate reader (BMG LABTEC, Ortenberg, German).

After quantification, equal protein quantities of cellular and EV protein samples (20 µg) were suspended in sample loading buffer (2-mecarptoethanol, glycerol, SDS, EDTA and bromophenol blue) and incubated at 95°C for 5 min. Samples were then separated by 10% sodium dodecyl sulfate polyacrylamide gel 19 electrophoresis (SDS-PAGE). Proteins were transferred to a 0.2 µm pore polyvinylidene difluoride (PVDF) membrane (Bio-Rad Laboratories, Hercules, California, United States) using a Trans-Blot®Turbo TM transfer system (Bio-Rad Laboratories, California, Hercules, United States). Membranes were then blocked using 1X Tris-buffered saline (TBS) containing 5% bovine serum albumin (BSA) and 0.1% Tween20 for 2h, at room temperature (RT). Subsequently, membranes were incubated overnight, at 4°C, with primary antibodies detailed in Supplementary Table 3. Membranes were incubated with a secondary anti-rabbit or anti-mouse antibody, depending on the primary antibody, during 1h, at RT. Finally, membranes were exposed to Clarity WB ECL substrate (Bio-Rad Laboratories, USA) and revealed using a ChemiDoc Imaging System (Bio-Rad Laboratories, USA).

- - 1. **Nanosight Tracking Analysis**

To assess the size and concentration of particles within our samples, we employed NTA using a Nanosight NS300 system equipped with a red laser (Malvern Panalytical, United Kingdom).

EV solutions were diluted in f-PBS1x and introduced into a 1 mL syringe. Five 30-second videos were recorded during the analysis, maintaining a continuous syringe pump flow rate of 3 units. Throughout the experiments, the temperature was maintained at a targeted 25°C. For all captures, a screen gain of 9 and a camera level of 10 were consistently applied. The obtained results were validated with a minimum of 1,000 total tracks and a particle count ranging between 10 and 50 per frame. The recorded videos were subsequently analyzed using NTA software version 3.4.

- - 1. **Transmission electron microscopy**

TEM was used to assess EV morphology, using negative staining. Firstly, 10 µL of EV samples were mounted on Formvar/carbon filmcoated mesh nickel grids (Electron Microscopy Sciences, Hatfield, PA, USA) and left standing for 2 min. Afterwards, 10 µL of 1% uranyl acetate were added onto the grids and excess liquid was removed. Visualization was carried out on a JEOL JEM 1400 microscope (Tokyo, Japan) at 120 kV. Images were recorded using the digital camera Orious 1100W (Tokyo, Japan).

- 1. **All-trans retinoic acid (ATRA) treatment**

NT2 cells were seeded in 5 150 mm Petri dishes with 20 ml of medium each, supplemented with EV-free FBS. ATRA was dissolved in DMSO, cells were treated with 10 µM ATRA every 2 days for 10 days, and conditioned medium was collected 72 hours after the 10 days of treatment (ATRA treatment schematic is depicted in Figure S1). A vehicle condition was always included, in which cells were treated with DMSO as a control. Cell differentiation was confirmed by a combination of cell morphology (as reported in [31]), real time quantitative PCR (RT-qPCR) for *POU5F1*, *NANOG*, *PAX6*, *SOX2*, *LIN28*, and *LIN28B,* as well as with western blot for NANOG and PAX6, always comparing vehicle versus ATRA-treated conditions. Also, miR-371a-3p, miR-372-3p, miR-373-3p and let-7e levels were assessed in both conditions.

- 1. **RNA extraction**

RNA from cell lines and tissues was extracted using TripleXtractor (Grisp, Portugal), as previously described in [32] and [33]. RNA from plasma samples and EV-derived RNA from all the different sample types was extracted using mirVana^TM^ miRNA isolation kit (ThermoFisher Scientific), following the manufacturer’s instructions. Before extraction, EV pellets were subjected to RNAse A treatment (ThermoFisher Scientific) for 30 minutes at 37ºC, followed by 30 minute incubation at 37ºC with RiboLock RNAse inhibitor (ThermoFisher Scientific) to inhibit the enzyme. RNA concentration and integrity were assessed after extraction using Invitrogen Qubit 4 Fluorometer (ThermoFisher Scientific).

- 1. **Messenger RNA RT-qPCR quantification in cells**

Levels of intracellular messenger RNA (mRNA) for *POU5F1*, *NANOG*, *PAX6*, *SOX2*, *LIN28* and *LIN28B* genes were measured in ATRA-treated and control NT2 cells through RT-qPCR. In brief, 1000 ng of cDNA were synthesized from RNA using the RevertAid cDNA Synthesis kit (Thermo Fisher Scientific) and RT-qPCR reaction was performed in QuantStudio 12K Flex platform (ThermoFisher Scientific), using Xpert Fast SYBR (Grisp, Portugal). β-Gus mRNA levels were also measured and used to normalize the results. The used primer sequences are presented in Supplementary Table 4.

- 1. **Reverse transcription, pre-amplification and microRNA testing**

RNA was reverse transcribed using TaqMan MicroRNA Reverse Transcription Kit (Applied Biosystems, USA), and quantitative real-time Polymerase Chain Reaction (RT-qPCR) was performed in QuantStudio 12K Flex platform (ThermoFisher Scientific), using Xpert Fast TaqMan Probe (Grisp). Pre-amplification reaction was performed for plasma and plasma-derived EV samples, using the TaqMan Preamp Master Mix (Thermo Fisher, 4391128). TaqMan™ microRNA assays for hsa-miR-371a-3p (assay ID 002124), hsa-miR-372–3p (assay ID 000560), hsa-miR-373–3p (assay ID 000561) and has-let-7e-5p (assay ID002406) were used for miRNA testing. RNU48 (assay ID 001006) was employed as endogenous quality control for cell lines and tissues, whereas miR-30b-5p (assay ID 000602) was used for plasmas and EV samples, as reported before in [27]. In the case of plasma circulating and EV-derived samples, miR-30-5p was only used as quality control, and miR-371-373 cluster and let-7e levels were represented as 40-Ct, as reported before [34]. Reactions were run in triplicate. RNA extracted from the seminoma-like cell line TCam-2 was plated as positive control, and a dilution series was included as an assessment of reaction efficiency. No template control (NTC) and no cDNA control were also included in all plates.

- 1. **Statistical analysis**

Comparisons between groups were performed using the non-parametric test Mann-Whitney U test, on GraphPad Software (GraphPad Software, Boston, MA, USA). P-values lower than 0.05 were considered statistically significant.

**Results**

- 1. **(T)GCT cells and tissues efficiently secrete EVs into culture media and plasma**

The seminoma-like TCam-2 cell line was the one which yielded most particles for both EV populations after calculating the secreted particle/cell ratio, whereas the ones that yielded the least were the JAR cells (Figures 1A and 1B). However, regarding absolute number of particles, the cell line that secreted the most according to the NTA analysis was 2102-Ep, an EC cell line, consistent for both isolated EV fractions (lEVs and sEVs), whereas the ones which secreted the least particles were JAR (CHC-like cell line) and JEG-3 (Figures S2A and S2B). Particle size mode was uniform across all cell lines (Figures S2C and S2D). lEV yielded significantly less particles than sEV in the samples (p=0.0003, Figure 1C), and overall showed significantly larger particle size than sEV (p<0.0001, Figure 1D).

Regarding RNA concentration, measured for all cell lines after EV-RNA extraction, sEV showed significantly higher RNA content than lEV (p=0.0405, Figure S3A). However, when calculating the RNA/particle ratio, no significant differences between the two isolated fractions were apparent (Figure S3B).

To further demonstrate proper EV isolation, and strictly following MISEV guidelines, western blot and TEM were also performed for EVs isolated from the different sample types: cell line-derived, tissue explant-derived and plasma-derived. The western blot (Figure 2A for cell line-derived and tissue explant-derived EVs, and Figure 2B for plasma) showed the presence of common EV markers, at least of two of the tested membrane-bound tetraspanins (CD9, CD63, CD81) and internal EV proteins (TSG101, HSC70, Alix), in both EV populations, for all sample types (Figures 2A and 2B). Also, there was under-representation of cytochrome-c, a common mitochondrial protein, in cell-derived and tissue explant-derived EVs when compared with the cell lysate (Figure 2A), whereas plasma-derived EVs showed the presence of albumin, the most common protein in plasma, as expected with the UC isolation (Figure 2B). Uncut western blots are represented in Figures S4 and S5.

Furthermore, spherical/cup-shaped vesicles were observed in TEM due to EV collapse and dehydration following sample preparation for the TEM analysis [35] (Figure 2C), visible across all EV sample types (TGCT cell lines, tissue and plasma EVs). Generally, the three sample types secreted a population of particles which were isolated into two different heterogeneous fractions of EVs (lEV and sEV), with protein content and particle morphology consistent with EV characterization, according to MISEV 2023 guidelines [30].

- 1. **MiR-371-373 cluster members are carried in (T)GCT cell line-derived EVs and their expression profiles mirror internal cellular miRNA levels**

RT-qPCR was performed in total RNA derived from (T)GCT cell lines, as well as from cell line-derived EVs released into conditioned media, separately for lEV and sEV populations.

The three miRNAs from the 371-373 cluster showed similar profiles in their cellular and EV-derived fractions (Figure 3A-I). 2102-Ep, an EC cell line, was the one with the highest levels across the three populations for the three miRNAs, whereas NOY-1, an ovarian YST cell line, was consistently the one with lower levels across all tested miRNAs and populations. The opposite was found for let-7e levels, with NOY-1 showing one of the highest levels of this miRNA in cells and EV-derived RNA, and 2102-Ep showing one of the lowest levels in the three studied fractions (Figure 3J-L). NCCIT and NT2 cell lines (EC cell lines, like 2102-Ep), also showed low levels of cellular let-7e (Figure 3J). Interestingly, NT2 cells were amongst the highest secreting cell lines for let-7e, both for lEV and sEV (Figure 3K-L). Overall, an opposite tendency was found between let-7e and the miR-371-373 cluster levels across the four analyzed miRNAs.

- 1. **ATRA treatment promotes cell differentiation in NT2 cells, shifting the cellular equilibrium of miR-371-373 and let-7e levels**

Due to this inverse correlation between miR-371-373 cluster and let-7e levels in the cell lines, and with the additional goal of assessing EV secretion in a cell line recapitulating TE features (i.e. more somatic-prone and less germ-like), the NT2 cell line was treated with ATRA, a known differentiating agent used before successfully in our group [31]. Differentiation was induced successfully with ATRA treatment over 10 days, confirmed on morphological assessment of cells (neuronal-like changes, Figure S6). Furthermore, these phenotypical changes were accompanied by a significant downregulation in transcript levels of pluripotency factors *NANOG*, *POU5F1* and *LIN28* (p=0.0087, p=0.0011 and p=0.0281, Figure 4A, 4B and 4E, respectively), as well as a decreasing tendency in *SOX2* and *LIN28B* (Figure 4C and 4F), on ATRA-treated cells. This was further supported by a significant upregulation of *PAX6* (p=0.004, Figure 4D). Decreased protein expression of NANOG and a slight increase in protein expression of PAX6 was also observed on ATRA-differentiated cells compared with the control, although not reaching statistical significance for PAX6 (Figure S7, and uncut western blots in Figure S8).

The levels of the stemness-related miRNAs of the 371-373 cluster were lower in ATRA-differentiated cells (Figure 5A-C), reaching statistical significance for miR-371a-3p, the most specific member of the cluster (p=0.0238, Figure 5A). On the other hand, ATRA-differentiated cells showed a significant increase in the levels of let-7e (p=0.0011, Figure 5D). Also, as stated previously, *LIN28*, inhibitor of let-7e family miRNA processing [36], exhibited a significant decrease in expression in ATRA-differentiated cells (Figure 4E).

- 1. **ATRA treatment inhibits secretion of miR-371-373 cluster members and promotes let-7e secretion in NT2-derived EVs, recapitulating cellular levels**

After successfully confirming differentiation of NT2 cells with ATRA, both fractions of EVs released by these cells into conditioned media were isolated through UC, from vehicle and treated conditions.

The secretion range (particles/cell) was higher in ATRA-treated cells for both EV fractions in comparison with the controls (Figure S9), although this was statistically significant only in the lEV fraction (p=0.0411, Figure S9A).

In the lEV fraction, miR-371a-3p and miR-372-3p decreased significantly in ATRA-treated cells (p=0.026 and p=0.026, Figures 6A and 6B, respectively). Of note, all the miR-371-373 cluster members displayed significant decreases in treated cells in the sEV population (p=0.0087, p=0.026 and p=0.0022, Figures 6E-G). Oppositely, let-7e levels showed a significant increase in treated cells on the lEV fraction (p=0.0043, Figure 6D), with no differences between conditions in the sEV fraction (Figure 6H).

- 1. **TGCT tissues release more EVs than adjacent unremarkable testicular parenchyma, and miR-371-373 cluster is upregulated in TGCT tissue-derived EVs**

Tissue pieces were freshly collected from TGCT patients immediately after surgery (orchiectomy or RPLND). The tissues were cultured in medium overnight, and EVs were isolated from the conditioned medium. The normalized number of secreted particles to the medium was significantly higher for tumor tissue when compared with unremarkable adjacent testicular parenchyma (testicular parenchyma that did not show signs of TGCT, GCNIS or other alterations), both for lEV and sEV (p=0.0005, Figure 7A and p=0.023, Figure 7B, respectively).

Globally, tumor-derived EVs presented higher RNA amount when compared to normal tissue EVs, both in large and small fractions (Figure S10). The cellular tissue levels of all miR-371-373 cluster members were significantly higher in non-TE TGCT tissues when compared with matched healthy testicular parenchyma (p=0.0002, p=0.0002 and p<0.0001, Figures S11A-C). Also, as expected [13, 27], TEs were negative for the miR-371-373 cluster, with significantly lower levels when compared with the non-TE TGCTs (p=0.022 for the three, Figures S11A-C). No significant differences were found for let-7e levels between the three groups (Figure S11D).

EVs were then isolated from the tissue explant-derived conditioned media by UC, and divided into lEV and sEV, recapitulating the cell line experiments. All miR-371-373 cluster members were significantly upregulated in EVs released from non-TE TGCT when compared with matched testicular parenchyma tissues, both for lEV (p=0.0043, p=0.0278 and p=0.0033, Figures 8A, 8C and 8E) and sEV (p=0.0001, p=0.0018 and p<0.0001, Figures 8B, 8D and 8F). TE-derived EVs showed lower miR-371-373 levels compared to non-TE TGCT and adjacent testicular parenchyma. Of note, patient TGCT9 presented a NS composed of 80% TE (along with EC - 11%; YST - 9%) and showed the lowest levels of EV-mir-371-373 cluster amongst the non-TE TGCTs tested, reflecting the high proportion of TE within the tumor. Contrarily, EV-let-7e levels were significantly lower in non-TE TGCTs comparing with adjacent testicular parenchyma tissue explant-derived EVs (p=0.0005 and p=0.0278, Figures 8G and 8H). Spearman correlation analysis for miR-371-373 cluster and let-7e cellular, lEV and sEV levels was performed. Cellular levels of the three members of the miR-371-373 cluster had moderate to strong positive correlation with their respective lEV and sEV secreted (conditioned medium)miRNA levels (Figure S12A-C). No significant correlation was found for let-7e, however (Figure S12D).

- 1. **miR-371-373 cluster and let-7e are carried in EVs in TGCT patient plasma samples, but plasma-derived EV-miR-371a-3p loses biomarker performance compared with circulating total miR-371a-3p**

Matched plasma samples to the previously tested tissue explants were then used for EV isolation, to enrich the sample for secreted miRNAs, and to subsequently assess the EV-levels of both miR-371-373 cluster and let-7e, with the aim to verify if there is improvement in the sensitivity of the test. The number of particles were similar between TGCT and HDs samples, both in lEVs and sEVs (Figure S13).

In plasma, a raw Ct approach (previously reported in [34]) was used to evaluate the results of plasma EV-microRNAs. The miR-371a-3p and miR-372-3p levels in the lEV population were significantly higher in non-TE TGCT group compared to the healthy donor (HD) group (p=0.013 and p=0.0399, Figure 9A and 9C). However, although we verified a tendency for higher levels of miR-371-373 cluster members in EVs overall, the discrimination towards levels in HD did not achieve significance. Looking at miR-371a-3p, the most specific member of the cluster (both in the clinical setting and in terms of chemical assay [27, 37, 38]), used as part of the IVD test in the clinic, levels were significantly higher in non-TE TGCT patients compared with HDs for the total circulating microRNA fraction (p=0.0017, Figure S14A), while for both matching EV-derived miRNA fractions there was amplification in the HD samples, showing a decrease in specificity of the assay (Figure 9A and 9B). As expected from the tissue explants results, the matched TE plasma samples were negative for miR-371-373 cluster (Figures 9A-F). However, contrary to cell lines and tissue explant EVs, plasma EV-derived let-7e showed no significant differences between the studied groups (Figure 9G and 9H). Spearman correlation analysis was performed (as with the tissue explants experiments), but there were no strong correlations between the total circulating and EV secreted levels for the studied miRNAs (Figure S15).

**Discussion**

MicroRNAs are non-coding RNAs that are part of the epigenetic regulation mechanisms of gene expression, playing significant roles both in ordinary biological processes and tumorigenesis [5, 39]. TGCTs are characterized by high levels of miR-371-373 [8, 19]. The cluster member miR-371a-3p has showed most promise as a sensitive and specific TGCT biomarker (being negative only in TE, the most differentiated form of TGCT), culminating in dedicated clinical trials and implementation as an IVD-approved test for clinical use [13, 40, 41]. To date, studies have investigated total circulating levels of miR-371-373; however, it remains elusive if these are carried inside EVs. Hence, in this study we aimed to characterize EVs from a wide range of TGCT samples, including cell lines, tissue explants and matched plasma samples from patients and healthy donors, and then use these samples to assess EV-microRNA expression (miR-371-373 cluster and let-7e).

EVs from both pre-clinical (*in vitro*) and clinical TGCT samples were successfully isolated by UC and characterized according to field standards [30], showing that both (T)GCT cell lines and tissues secrete EVs into culture medium and plasma. Furthermore, in our study, all EV-RNA samples were subjected to a pre-extraction RNAse step treatment, to assure that the population of miRNAs analyzed originates inside the EVs [42, 43].

Herein, we used both lEV and sEV from the UC to investigate differences in miRNA cargo between the two populations. Some previous studies report differences in microRNA cargo among EV fractions, particularly in sEVs (which are more enriched in exosomes) [44, 45]. In our study, we did not observe remarkable differences between these two fractions for the levels of analyzed miRNAs, which were homogeneous and recapitulated their intrinsic cellular levels. Other EV isolation methods could be assessed in the future depending on the objective, such as size exclusion chromatography (SEC), sucrose cushion UC, microfluidic devices or antibody-coated magnetic beads [46], each showing potential advantages and disadvantages [47]. Importantly, our experimental protocols were designed in accordance with MISEV guidelines, which aim to list a series of minimal requirements for pursuing EV research and therefore minimize variability among studies in the field [30]. As the target miRNA of interest is already known in our study, simpler approaches that allow for a maximum recovery of the target of interest are ideal. However, for functional studies on a specific EV population, it may be argued that SEC or sucrose cushion UC could yield a purer EV fraction [30]. Also, a limited number of cases were analyzed. However, the study gains granularity by including pre-clinical samples representative of a vast array of TGCT phenotypes, as well as matched clinical samples collected from the same patients, allowing to establish proper comparisons and conclude on secretion of EVs [59].

For proper advance of GCT translational research, biobanks and relevant sets of pre-clinical and clinical samples are needed, involving collaboration between clinical and research staff [48]. This is particularly important in EV-related research, in which fresh tissue explants and liquid biopsies should be preserved with the most caution in order to standardize conditions and avoid compromising study integrity [49]. Thus, an optimized pipeline involving an interdepartmental circuit between clinicians, pathologists and researchers was ultimately established in the present study, to successfully collect and process EV samples from different types of samples (tissue and matched plasmas from the same patients at orchiectomy and RPLND time). This workflow allowed for establishing a comparison between levels of EVs and miRNA cargo across histological subtypes represented in the different cell lines, tissue explants and matched plasma samples, showing that miR-371-373 cluster may be indeed secreted into TGCT EVs, and that the EV-derived miRNA profile mostly mirrors the internal cellular miRNA levels, in both cell lines and clinical samples (showing moderate to strong positive correlation in tissue cellular and EV-secreted miRNA levels). TE samples presented a clear downregulation of EV-miR-371-373, as also occurs with cellular levels. In the future, this ongoing effort will also allow for additional TGCT-EV studies, to further understand the role of these nano-sized particles in GCT cell communication and signaling and for potential EV biomarker-driven studies.

Tumor tissues are reported to secrete a higher number of EV particles when compared with healthy tissues [50-52]. This was verified in our study with the tissue explant experiments. However, the same was not observed in our plasma-derived EVs, in which the number of particles of TGCT patients and HDs did not show significant variation. This higher number of EV particles released has been correlated with tumor staging and volume of disease as measurements of disease burden [53], and the TGCT patients included in this study are predominantly stage I patients, which could in part explain these findings. Also, specific demographic and health characteristics of the selected HD population may contribute to this observation.

While all previous studies investigating miR-371a-3p as a diagnostic and monitoring biomarker in the clinic have focused on the total circulating miRNA fraction [11, 13, 14, 16, 17], it remains elusive to date how focusing on the EV-derived miRNA fraction would affect the test performance. In other tumor models, studies are beginning to report diagnostic performance of biomarkers quantified specifically from EV cargo, with variable results [54-56]. We aimed to answer this question by comparing the “classical” total RNA pipeline with EV-derived miR-371a-3p in patient samples. Despite significantly higher levels of miR-371a-3p and miR-372-3p in lEVs of TGCT patients versus HDs, our results support that focusing on plasma-derived EV-miR-371a-3p may overall result on a decrease in biomarker performance. This drop in specificity of the assay, when combined with the considerably higher volume of plasma required to efficiently isolate EVs through UC (2 mL) and the more time-consuming protocol of EV isolation prior to RNA extraction, make this approach likely unfit to use in the clinical practice, at the present time. One of the issues identified in the plasma-derived EV-RNA was some background in the HD samples, not seen in the total circulating miRNA fraction. We hypothesize that the preamplification step before RT-qPCR measurement, in conjunction with the high volume of plasma needed to isolate the EVs, could contribute to this issue [18, 57]. An analysis by droplet digital PCR (ddPCR) could be useful in avoiding this issue, since previous ddPCR protocols do not rely on preamplification steps [18, 58]. Furthermore, this is the first study in which UC isolated EVs are used to analyze TGCT-derived miR-371-373 cluster levels in clinical samples, and these methodologies will surely require further refining, such as better cutoff definition based on larger patient cohorts.

Previous biological studies have demonstrated that non-TE TGCTs show downregulation of the tumor suppressor let-7 family of miRNAs [20]. This opposing miR-371-373 upregulation / let-7 downregulation relationship is supported by expression of the pluripotency factor *LIN28*, highly expressed in SE and GCNIS, which downstream negatively regulates the expression of let-7 miRNA family members expression [20, 21, 59, 60]. After treatment with ATRA, known to induce differentiation of embryonal carcinoma NT2 cells [31], *LIN28* expression was significantly downregulated, as it happened with pluripotency factors *NANOG* and *POU5F1*, with the opposite occurring for *PAX6,* a marker of differentiation. Importantly, let-7e (the most downregulated let-7 family miRNA, negatively associated with *MYCN* and *LIN28* in GCTs [20]) was significantly upregulated. This result supports a negative feedback mechanism between miR-371-373 cluster and let-7 family miRNAs in GCT stepwise differentiation from pluripotent phenotypes (such as SE or EC) to more somatic-prone ones (like the TE), requiring additional functional studies for confirmation. Moreover, the same shift in microRNA profiles was seen in EV-derived RNA, as miR-371-373 cluster levels decreased significantly in EVs from ATRA-treated cells when compared with control ones, with the opposite verified for EV-derived let-7e. Finally, this was also observed in the patient samples, as pure TE samples not only showed absence of miR-371-373 cluster intrinsically, but also on the EVs they secreted, when compared with non-TE TGCTs. Being the least specific member of the cluster, miR-372-3p showed some PCR amplification in these samples, which is associated with chemical background noise from the assay [27]. EVs are known to contain a diverse array of cargo, including miRNAs, that can be transferred to other cells and induce phenotypic changes, being involved in processes such as pregnancy, embryonic development and cell differentiation and fate commitment [61-63]. In our integrated model composed of cell lines, patient-derived tissue explants and plasma samples, upon differentiation (recapitulating differentiation from EC to TE), we verify a halt to the secretion of miR-371-373 in EVs, accompanied by an increase in EV-let-7e secretion in the *in vitro* experiments. Overall, the fact that this miRNA profile shift is observed in EV-derived RNA suggests that this biological process is relevant for cells to undergo phenotypic changes, needing to protect and encapsulate these miRNAs in EVs, to successfully use them to communicate the “message” to other cells, either in the local tumor microenvironment or at distance.

In this context, our results suggest an upregulation in TGCT-EVs’ miR-371-373 cluster levels, with recent data supporting this and suggesting that EVs derived from TGCT cell lines can deliver miRNAs into cells of the tumor microenvironment, with the potential of contributing to tumor progression [26]. Animal model studies could be a vital resource to further expand our understanding of EVs and their cargo in tumor progression and formation of pre-metastatic niche, in which EVs have been implicated [52, 64, 65]. Studying EVs in well-established GCT animal models, such as germ cell-specific *Pten* and *Kras* (gPAK) mutant mice [66, 67], will be essential to further understand the role of miR-371-373 cluster and let-7 family members in TGCT cell communication and biology. It has been recently shown that embryonal carcinoma cells in the gPAK model express and secrete miRNAs of the miR-290-295 cluster, the mouse orthologs of human miR-371-373 [68]. Exploring EV-derived miRNA functions in a mouse model with an intact immune system and representative tumor microenvironment holds promise for shedding light on how miRNA transfer via EVs influence TGCT pathogenesis.

In conclusion, our study showed that EVs from TGCT cell lines and clinical sample depict high levels of miR-371-373 cluster, mirroring their cellular quantities, with a clear shift in the EV-miRNA levels with differentiation, showing absence of these miRNAs in TE. To our knowledge, this is the first study validating that TGCT-derived EVs from relevant clinical samples (matched tissue explants and plasma) indeed carry high levels of miR-371-373 cluster, suggesting there may be an EV-based communication mechanism with these miRNAs from the tissue to the plasma. A microRNA switch (see Graphical Abstract) is seen upon cell differentiation induced with ATRA, which triggers downregulation in the pluripotency-related miR-371-373 cluster, downregulation of *LIN28* and other pluripotency-related markers, and upregulation of let-7e [69]. Consequently, although biomarker performance suggests that EV-miR-371a-3p may not be the best candidate to use in the clinic when compared with total circulating miR-371a-3p, the observed changes in the miRNA cargo of EVs highlights their utility as indicators of TGCT cell state and mediators of intercellular communication. Furthermore, these EVs may be used in the future as therapeutic targets, as they are suggested to deliver oncogenic cargo to near and distant tumor microenvironment cells [26]. The pipeline and consequent results herein presented could be used in the future for the discovery of novel clinical EV-derived biomarkers and to deepen our understanding in TGCT biology.

**Declarations:**

**Ethics approval and consent to participate**

Samples were collected after obtaining patient informed consent and ethics approval (CES176/022)

**Consent for publication**

Not applicable

**Availability of data and materials**

Not applicable

**Competing interests**

The authors declare that they have no competing interests

**Funding**

NTT and CL hold PhD fellowships from Fundação para a Ciência e Tecnologia (FCT) - 2022.09566.BD and 2021.06731.BD respectively, and ML was supported by a Genomics Scholar Award from the Cornell University Center for Vertebrate Genomics. The authors would like to acknowledge the funding from miREpiTestis – ‘Unveiling miR-371-373 cluster epigenetic reprogramming and downstream targets in testicular germ cell tumors’ (PI190-CI-IPOP-23-2023) and the Sandra Atlas Bass Endowment for Cancer Research to RSW.

**Authors' contributions**

NTT wrote the manuscript. NTT, CL, VC and FFP performed the experiments. RSS provided help with liquid biopsy and tissues processing. IB and JM provided clinical information. ML and RW provided scientific input on let-7 experiments. RH, AB, CJ and JL supervised the activities. JL supervised the research project.

**References**

1. Bray, F., M. Laversanne, H. Sung, et al., *Global cancer statistics 2022: GLOBOCAN estimates of incidence and mortality worldwide for 36 cancers in 185 countries.* CA Cancer J Clin, 2024. **74**(3): p. 229-263. 10.3322/caac.21834

2. Tavares, N.T., R. Henrique, A. Bagrodia, C. Jeronimo, and J. Lobo, *A stroll through the present and future of testicular germ cell tumour biomarkers.* Expert Rev Mol Diagn, 2023. **23**(5): p. 405-418. 10.1080/14737159.2023.2206956

3. Almstrup, K., J. Lobo, N. Morup, et al., *Application of miRNAs in the diagnosis and monitoring of testicular germ cell tumours.* Nat Rev Urol, 2020. **17**(4): p. 201-213. 10.1038/s41585-020-0296-x

4. Oosterhuis, J.W. and L.H.J. Looijenga, *Human germ cell tumours from a developmental perspective.* Nat Rev Cancer, 2019. **19**(9): p. 522-537. 10.1038/s41568-019-0178-9

5. Iorio, M.V. and C.M. Croce, *MicroRNAs in cancer: small molecules with a huge impact.* J Clin Oncol, 2009. **27**(34): p. 5848-56. 10.1200/JCO.2009.24.0317

6. Peng, Y. and C.M. Croce, *The role of MicroRNAs in human cancer.* Signal Transduct Target Ther, 2016. **1**: p. 15004. 10.1038/sigtrans.2015.4

7. Constancio, V., N.T. Tavares, R. Henrique, C. Jeronimo, and J. Lobo, *MiRNA biomarkers in cancers of the male reproductive system: Are we approaching clinical application?* Andrology, 2023. **11**(4): p. 651-667. 10.1111/andr.13258

8. Murray, M.J., D.J. Halsall, C.E. Hook, D.M. Williams, J.C. Nicholson, and N. Coleman, *Identification of microRNAs From the miR-371~373 and miR-302 clusters as potential serum biomarkers of malignant germ cell tumors.* Am J Clin Pathol, 2011. **135**(1): p. 119-25. 10.1309/AJCPOE11KEYZCJHT

9. Bailey, S., M. Ferraresso, L. Alonso-Crisostomo, et al., *Targeting oncogenic microRNAs from the miR-371~373 and miR-302/367 clusters in malignant germ cell tumours causes growth inhibition through cell cycle disruption.* Br J Cancer, 2023. **129**(9): p. 1451-1461. 10.1038/s41416-023-02453-1

10. Tavares, N.T., J. Lobo, and A. Bagrodia, *MicroRNAs for detecting occult genitourinary cancer.* Curr Opin Urol, 2024. **34**(1): p. 20-26. 10.1097/MOU.0000000000001137

11. Badia, R.R., D. Abe, D. Wong, et al., *Real-World Application of Pre-Orchiectomy miR-371a-3p Test in Testicular Germ Cell Tumor Management.* J Urol, 2021. **205**(1): p. 137-144. 10.1097/JU.0000000000001337

12. Conduit, C., P.S. Grimison, J.H. Lewin, et al., *CLIMATE: Assessing the clinical utility of miR-371a-3p as a marker of residual disease in stage 1 testicular germ cell tumour (TGCT) following orchiectomy: ANZUP 1906.* Journal of Clinical Oncology, 2023. **41**(16_suppl): p. TPS5100-TPS5100. 10.1200/JCO.2023.41.16_suppl.TPS5100

13. Dieckmann, K.P., A. Radtke, L. Geczi, et al., *Serum Levels of MicroRNA-371a-3p (M371 Test) as a New Biomarker of Testicular Germ Cell Tumors: Results of a Prospective Multicentric Study.* J Clin Oncol, 2019. **37**(16): p. 1412-1423. 10.1200/JCO.18.01480

14. Fankhauser, C.D., A.J. Christiansen, C. Rothermundt, et al., *Detection of recurrences using serum miR-371a-3p during active surveillance in men with stage I testicular germ cell tumours.* Br J Cancer, 2022. **126**(8): p. 1140-1144. 10.1038/s41416-021-01643-z

15. Leao, R., T. van Agthoven, A. Figueiredo, et al., *Serum miRNA Predicts Viable Disease after Chemotherapy in Patients with Testicular Nonseminoma Germ Cell Tumor.* J Urol, 2018. **200**(1): p. 126-135. 10.1016/j.juro.2018.02.068

16. Lobo, J., R. Leao, A.J.M. Gillis, et al., *Utility of Serum miR-371a-3p in Predicting Relapse on Surveillance in Patients with Clinical Stage I Testicular Germ Cell Cancer.* Eur Urol Oncol, 2021. **4**(3): p. 483-491. 10.1016/j.euo.2020.11.004

17. Nappi, L., M. Thi, A. Lum, et al., *Developing a Highly Specific Biomarker for Germ Cell Malignancies: Plasma miR371 Expression Across the Germ Cell Malignancy Spectrum.* J Clin Oncol, 2019. **37**(33): p. 3090-3098. 10.1200/JCO.18.02057

18. Sequeira, J.P., J. Lobo, V. Constancio, et al., *DigiMir Test: Establishing a Novel Pipeline for MiR-371a Quantification Using Droplet Digital PCR in Liquid Biopsies From Testicular Germ Cell Tumor Patients.* Front Oncol, 2022. **12**: p. 876732. 10.3389/fonc.2022.876732

19. Dieckmann, K.P., C. Dumlupinar, F. Grobelny, et al., *Testicular neoplasms: the interrelationships of serum levels of microRNA-371a-3p (M371) and classical tumor markers with histology, clinical staging, and age-a statistical analysis.* J Cancer Res Clin Oncol, 2023. **149**(10): p. 7079-7090. 10.1007/s00432-023-04664-8

20. Murray, M.J., H.K. Saini, C.A. Siegler, et al., *LIN28 Expression in malignant germ cell tumors downregulates let-7 and increases oncogene levels.* Cancer Res, 2013. **73**(15): p. 4872-84. 10.1158/0008-5472.CAN-12-2085

21. West, J.A., S.R. Viswanathan, A. Yabuuchi, et al., *A role for Lin28 in primordial germ-cell development and germ-cell malignancy.* Nature, 2009. **460**(7257): p. 909-13. 10.1038/nature08210

22. van Niel, G., G. D'Angelo, and G. Raposo, *Shedding light on the cell biology of extracellular vesicles.* Nat Rev Mol Cell Biol, 2018. **19**(4): p. 213-228. 10.1038/nrm.2017.125

23. Lourenco, C., V. Constancio, R. Henrique, A. Carvalho, and C. Jeronimo, *Urinary Extracellular Vesicles as Potential Biomarkers for Urologic Cancers: An Overview of Current Methods and Advances.* Cancers (Basel), 2021. **13**(7). 10.3390/cancers13071529

24. Dai, J., Y. Jiang, H. Hu, S. Zhang, and Y. Chen, *Extracellular vesicles as modulators of glioblastoma progression and tumor microenvironment.* Pathol Oncol Res, 2024. **30**: p. 1611549. 10.3389/pore.2024.1611549

25. Groot, M. and H. Lee, *Sorting Mechanisms for MicroRNAs into Extracellular Vesicles and Their Associated Diseases.* Cells, 2020. **9**(4). 10.3390/cells9041044

26. Alonso-Crisostomo, L., J. Trendell, M. Ferraresso, et al., *Testicular germ cell tumour cells release microRNA-containing extracellular vesicles that induce phenotypic and genotypic changes in cells of the tumour microenvironment.* Int J Cancer, 2024. **154**(2): p. 372-388. 10.1002/ijc.34697

27. Lobo, J., A.J.M. Gillis, A. van den Berg, et al., *Identification and Validation Model for Informative Liquid Biopsy-Based microRNA Biomarkers: Insights from Germ Cell Tumor In Vitro, In Vivo and Patient-Derived Data.* Cells, 2019. **8**(12). 10.3390/cells8121637

28. Lobo, J., A.L. Costa, B. Vilela-Salgueiro, et al., *Testicular germ cell tumors: revisiting a series in light of the new WHO classification and AJCC staging systems, focusing on challenges for pathologists.* Hum Pathol, 2018. **82**: p. 113-124. 10.1016/j.humpath.2018.07.016

29. Hoshino, A., H.S. Kim, L. Bojmar, et al., *Extracellular Vesicle and Particle Biomarkers Define Multiple Human Cancers.* Cell, 2020. **182**(4): p. 1044-1061 e18. 10.1016/j.cell.2020.07.009

30. Welsh, J.A., D.C.I. Goberdhan, L. O'Driscoll, et al., *Minimal information for studies of extracellular vesicles (MISEV2023): From basic to advanced approaches.* J Extracell Vesicles, 2024. **13**(2): p. e12404. 10.1002/jev2.12404

31. Lobo, J., A.R. Cardoso, V. Miranda-Goncalves, et al., *Targeting Germ Cell Tumors with the Newly Synthesized Flavanone-Derived Compound MLo1302 Efficiently Reduces Tumor Cell Viability and Induces Apoptosis and Cell Cycle Arrest.* Pharmaceutics, 2021. **13**(1). 10.3390/pharmaceutics13010073

32. Miranda-Goncalves, V., J. Lobo, C. Guimaraes-Teixeira, et al., *The component of the m(6)A writer complex VIRMA is implicated in aggressive tumor phenotype, DNA damage response and cisplatin resistance in germ cell tumors.* J Exp Clin Cancer Res, 2021. **40**(1): p. 268. 10.1186/s13046-021-02072-9

33. Silva-Santos, R.M., P. Costa-Pinheiro, A. Luis, et al., *MicroRNA profile: a promising ancillary tool for accurate renal cell tumour diagnosis.* Br J Cancer, 2013. **109**(10): p. 2646-53. 10.1038/bjc.2013.552

34. Lafin, J.T., C.G. Scarpini, A. Amini, et al., *Refining the serum miR-371a-3p test for viable germ cell tumor detection.* Sci Rep, 2023. **13**(1): p. 10558. 10.1038/s41598-023-37271-1

35. Thery, C., S. Amigorena, G. Raposo, and A. Clayton, *Isolation and characterization of exosomes from cell culture supernatants and biological fluids.* Curr Protoc Cell Biol, 2006. **Chapter 3**: p. Unit 3 22. 10.1002/0471143030.cb0322s30

36. Newman, M.A., J.M. Thomson, and S.M. Hammond, *Lin-28 interaction with the Let-7 precursor loop mediates regulated microRNA processing.* RNA, 2008. **14**(8): p. 1539-49. 10.1261/rna.1155108

37. Leao, R., M. Albersen, L.H.J. Looijenga, et al., *Circulating MicroRNAs, the Next-Generation Serum Biomarkers in Testicular Germ Cell Tumours: A Systematic Review.* Eur Urol, 2021. **80**(4): p. 456-466. 10.1016/j.eururo.2021.06.006

38. Tavares, N.T., R. Henrique, C. Jeronimo, and J. Lobo, *Current Role of MicroRNAs in the Diagnosis and Clinical Management of Germ Cell Tumors.* Surg Pathol Clin, 2025. **18**(1): p. 91-100. 10.1016/j.path.2024.08.003

39. Pasquinelli, A.E., B.J. Reinhart, F. Slack, et al., *Conservation of the sequence and temporal expression of let-7 heterochronic regulatory RNA.* Nature, 2000. **408**(6808): p. 86-9. 10.1038/35040556

40. Alsyouf, M., L. Nappi, C. Nichols, and S. Daneshmand, *Plasma Micro-RNA 371 Expression in Early-Stage Germ Cell Tumors: Are We Ready to Move Toward Biology-Based Decision Making?* J Clin Oncol, 2023. **41**(14): p. 2478-2482. 10.1200/JCO.22.02002

41. Pycha, S., C. D'Elia, C. Mian, et al., *Evaluation of the M371-Test Under Real-life Conditions for Diagnosis and Follow Up of Testicular Germ Cell Tumors.* Anticancer Res, 2023. **43**(4): p. 1649-1653. 10.21873/anticanres.16316

42. Bracht, J.W.P., A. Gimenez-Capitan, C.Y. Huang, et al., *Analysis of extracellular vesicle mRNA derived from plasma using the nCounter platform.* Sci Rep, 2021. **11**(1): p. 3712. 10.1038/s41598-021-83132-0

43. Teixeira-Marques, A., S. Monteiro-Reis, D. Montezuma, et al., *Improved recovery of urinary small extracellular vesicles by differential ultracentrifugation.* Sci Rep, 2024. **14**(1): p. 12267. 10.1038/s41598-024-62783-9

44. Siwaponanan, P., P. Kaewkumdee, W. Phromawan, et al., *Increased expression of six-large extracellular vesicle-derived miRNAs signature for nonvalvular atrial fibrillation.* J Transl Med, 2022. **20**(1): p. 4. 10.1186/s12967-021-03213-6

45. Mao, Y., M. Zhang, L. Wang, Y. Lu, X. Hu, and Z. Chen, *Role of microRNA carried by small extracellular vesicles in urological tumors.* Front Cell Dev Biol, 2023. **11**: p. 1192937. 10.3389/fcell.2023.1192937

46. De Sousa, K.P., I. Rossi, M. Abdullahi, M.I. Ramirez, D. Stratton, and J.M. Inal, *Isolation and characterization of extracellular vesicles and future directions in diagnosis and therapy.* Wiley Interdiscip Rev Nanomed Nanobiotechnol, 2023. **15**(1): p. e1835. 10.1002/wnan.1835

47. Brennan, K., K. Martin, S.P. FitzGerald, et al., *A comparison of methods for the isolation and separation of extracellular vesicles from protein and lipid particles in human serum.* Sci Rep, 2020. **10**(1): p. 1039. 10.1038/s41598-020-57497-7

48. Fonseca, A., J. Lobo, F.K. Hazard, et al., *Advancing clinical and translational research in germ cell tumours (GCT): recommendations from the Malignant Germ Cell International Consortium.* Br J Cancer, 2022. **127**(9): p. 1577-1583. 10.1038/s41416-022-02000-4

49. Gandham, S., X. Su, J. Wood, et al., *Technologies and Standardization in Research on Extracellular Vesicles.* Trends Biotechnol, 2020. **38**(10): p. 1066-1098. 10.1016/j.tibtech.2020.05.012

50. Lazaro-Ibanez, E., A. Sanz-Garcia, T. Visakorpi, et al., *Different gDNA content in the subpopulations of prostate cancer extracellular vesicles: apoptotic bodies, microvesicles, and exosomes.* Prostate, 2014. **74**(14): p. 1379-90. 10.1002/pros.22853

51. Osti, D., M. Del Bene, G. Rappa, et al., *Clinical Significance of Extracellular Vesicles in Plasma from Glioblastoma Patients.* Clin Cancer Res, 2019. **25**(1): p. 266-276. 10.1158/1078-0432.CCR-18-1941

52. Becker, A., B.K. Thakur, J.M. Weiss, H.S. Kim, H. Peinado, and D. Lyden, *Extracellular Vesicles in Cancer: Cell-to-Cell Mediators of Metastasis.* Cancer Cell, 2016. **30**(6): p. 836-848. 10.1016/j.ccell.2016.10.009

53. Baran, J., M. Baj-Krzyworzeka, K. Weglarczyk, et al., *Circulating tumour-derived microvesicles in plasma of gastric cancer patients.* Cancer Immunol Immunother, 2010. **59**(6): p. 841-50. 10.1007/s00262-009-0808-2

54. Endzelins, E., A. Berger, V. Melne, et al., *Detection of circulating miRNAs: comparative analysis of extracellular vesicle-incorporated miRNAs and cell-free miRNAs in whole plasma of prostate cancer patients.* BMC Cancer, 2017. **17**(1): p. 730. 10.1186/s12885-017-3737-z

55. Armstrong, D.A., B.B. Green, J.D. Seigne, A.R. Schned, and C.J. Marsit, *MicroRNA molecular profiling from matched tumor and bio-fluids in bladder cancer.* Mol Cancer, 2015. **14**: p. 194. 10.1186/s12943-015-0466-2

56. Dong, L., C. Hu, Z. Ma, et al., *Urinary extracellular vesicle-derived miR-126-3p predicts lymph node invasion in patients with high-risk prostate cancer.* Med Oncol, 2024. **41**(7): p. 169. 10.1007/s12032-024-02400-x

57. Christiansen, A.J., J. Lobo, C.D. Fankhauser, et al., *Impact of differing methodologies for serum miRNA-371a-3p assessment in stage I testicular germ cell cancer recurrence.* Front Oncol, 2022. **12**: p. 1056823. 10.3389/fonc.2022.1056823

58. Myklebust, M.P., A. Thor, B. Rosenlund, et al., *Serum miR371 in testicular germ cell cancer before and after orchiectomy, assessed by digital-droplet PCR in a prospective study.* Sci Rep, 2021. **11**(1): p. 15582. 10.1038/s41598-021-94812-2

59. Cao, D., R.W. Allan, L. Cheng, et al., *RNA-binding protein LIN28 is a marker for testicular germ cell tumors.* Hum Pathol, 2011. **42**(5): p. 710-8. 10.1016/j.humpath.2010.09.007

60. Gillis, A.J., H. Stoop, K. Biermann, et al., *Expression and interdependencies of pluripotency factors LIN28, OCT3/4, NANOG and SOX2 in human testicular germ cells and tumours of the testis.* Int J Androl, 2011. **34**(4 Pt 2): p. e160-74. 10.1111/j.1365-2605.2011.01148.x

61. Hur, Y.H., R.A. Cerione, and M.A. Antonyak, *Extracellular vesicles and their roles in stem cell biology.* Stem Cells, 2020. **38**(4): p. 469-476. 10.1002/stem.3140

62. Kurian, N.K. and D. Modi, *Extracellular vesicle mediated embryo-endometrial cross talk during implantation and in pregnancy.* J Assist Reprod Genet, 2019. **36**(2): p. 189-198. 10.1007/s10815-018-1343-x

63. Katsman, D., E.J. Stackpole, D.R. Domin, and D.B. Farber, *Embryonic stem cell-derived microvesicles induce gene expression changes in Muller cells of the retina.* PLoS One, 2012. **7**(11): p. e50417. 10.1371/journal.pone.0050417

64. Hoshino, A., B. Costa-Silva, T.L. Shen, et al., *Tumour exosome integrins determine organotropic metastasis.* Nature, 2015. **527**(7578): p. 329-35. 10.1038/nature15756

65. Peinado, H., M. Aleckovic, S. Lavotshkin, et al., *Melanoma exosomes educate bone marrow progenitor cells toward a pro-metastatic phenotype through MET.* Nat Med, 2012. **18**(6): p. 883-91. 10.1038/nm.2753

66. Lyndaker, A.M., T.M. Pierpont, A.R. Loehr, and R.S. Weiss, *A Genetically Engineered Mouse Model of Malignant Testicular Germ Cell Tumors.* Methods Mol Biol, 2021. **2195**: p. 147-165. 10.1007/978-1-0716-0860-9_11

67. Pierpont, T.M., A.M. Lyndaker, C.M. Anderson, et al., *Chemotherapy-Induced Depletion of OCT4-Positive Cancer Stem Cells in a Mouse Model of Malignant Testicular Cancer.* Cell Rep, 2017. **21**(7): p. 1896-1909. 10.1016/j.celrep.2017.10.078

68. Loehr, A.R., D.M. Timmerman, M. Liu, et al., *Analysis of a mouse germ cell tumor model establishes pluripotency-associated miRNAs as conserved serum biomarkers for germ cell cancer detection.* Sci Rep, 2025. **15**(1): p. 4452. 10.1038/s41598-025-88554-8

69. Melton, C., R.L. Judson, and R. Blelloch, *Opposing microRNA families regulate self-renewal in mouse embryonic stem cells.* Nature, 2010. **463**(7281): p. 621-6. 10.1038/nature08725

**Figure legends:**

**Figure 1:** Secretion range (particles/cell) for the separate cell lines in lEV (A) and sEV (B); particle number/ml (C) and particle size (D) for both EV populations in all basal cell line experiments. Data shown as mean ± SEM for 3 independent experiments. *** - p < 0.001, **** - p < 0.0001.

**Figure 2:** EV characterization by western blot for traditional EV markers and cellular contaminants in representative cell line and tissue-derived EVs (A) and plasma-derived EVs (B), and TEM imaging (C) for TGCT cell lines (C1, C2 for TCam-2 lEV and sEV and C3,C4 for JEG-3 lEV and sEV), tissue (C5,C6) and plasma (C7,C8) EVs. Higher exposure time had to be used for plasma EV samples western blot (B) when compared with A.

**Figure 3:** MicroRNA levels for miR-371a-3p, miR-372-3p, miR-373-3p and let-7e in (T)GCT cell lines’ internal levels (A,D,G,J), and respective large (B,E,H,K) and small EVs (C,F,I,L). Data shown as mean ± SEM for 3 independent experiments.

**Figure 4:** Pluripotency and differentiation-related genes expression levels for NT2 cells treated with ATRA (A – *NANOG*, B – *POU5F1*, C – *SOX2*, D – *PAX6*, E – *LIN28*, F – *LIN28B*), compared with vehicle controls. Data shown as mean ± SEM for 5 independent experiments; * - p < 0.05, ** - p < 0.01.

**Figure 5:** MiR-371-373 cluster (A,B,C) and let-7e (D) levels for NT2 cells treated with ATRA, compared with vehicle-treated controls. Data shown as mean ± SEM for 5 independent experiments; * - p < 0.05, ** - p < 0.01.

**Figure 6:** MiR-371-373 cluster and let-7e EV-derived levels for NT2 cells treated with ATRA, compared with vehicle-treated controls, for both EV isolated populations (lEV in A-D, sEV in E-H). Data shown as mean ± SEM for 5 independent experiments; * - p < 0.05, ** - p < 0.01.

**Figure 7:** Normalized number of particles secreted in TGCT tissue and adjacent testicular parenchyma, for lEV (A) and sEV (B). Data shown as mean ± SEM; * - p < 0.05, *** - p < 0.001.

**Figure 8:** EV-derived microRNA levels for miR-371a-3p (A,B), miR-372-3p (C,D), miR-373-3p (E,F) and let-7e (G,H), in non-TE TGCT, TE and adjacent testicular parenchyma tissue explants, for lEV and sEV. Data shown on a log scale as mean ± SEM; * - p < 0.05, ** - p < 0.01, *** - p < 0.001, **** - p < 0.0001.

**Figure 9:** EV-derived microRNA levels (40 - Ct) for miR-371a-3p (A,B), miR-372-3p (C,D), miR-373-3p (E,F) and let-7e (G,H), in non-TE TGCT, TE and HD plasma samples, for lEV and sEV. Data shown as mean ± SEM; * - p < 0.05.

**Supplementary information:**

**Supplementary Table 1:** (T)GCT cell lines used in the study.

| **Cell line** | **Histological type – tissue of origin** | **Cell culture medium** |
| --- | --- | --- |
| TCam-2 | Testicular seminoma | RPMI |
| NCCIT | Mediastinal embryonal carcinoma (pluripotent) | DMEM |
| 2102-Ep | Testicular embryonal carcinoma | DMEM |
| H12.1 | TGCT, with embryonal carcinoma | DMEM |
| NTERA-2 (NT2) | Testicular embryonal carcinoma (pluripotent) | DMEM |
| NOY-1 | Ovarian yolk sac tumor | RPMI |
| JEG-3 | Testicular choriocarcinoma | DMEM |
| JAR | Trophoblastic tumor of the placenta, with choriocarcinoma features | DMEM |
| 1777Met | Retroperitoneal lymph node metastasis, with embryonal carcinoma features | DMEM |

**Supplementary Table 2:** Clinical and histopathological parameters of the patients included in the study. Arrows (⇑) represent abnormal elevation of the classical serum tumor markers. Asterisks (*) represent the histological components assessed in the evaluated tissue specimens for mixed NS GCTs.

| **Sample** | **Tumor size (cm)** | **Cultured tissue fragment size (cm)** | **Age** | **[B-HCG]** | **[LDH]** | **[AFP]** | **Histological Type** | **Staging (pTNM)** |
| --- | --- | --- | --- | --- | --- | --- | --- | --- |
| TGCT1 | 4.5 x 3 x 3 | 2.2 x 2.3 x 0.4 | 40 | ⇑266 | ⇑832 | 3.87 | NS, Mixed (EC - 90%*; YST - 10%) | pT2N3M1a (III) |
| TGCT2 | 3.6 x 2.5 | 2.2 x 1.5 x 0.3 | 26 | 2 | 131 | 1.9 | NS, Mixed (EC - 95%*; SE - 5%) | pT2N0M0 (I) |
| TGCT3 | 6 x 5 | 4.0 x 1.5 x 0.5 | 38 | 8.4 | 236 | 2.7 | SE | pT2cN1M0 (II) |
| TGCT4 | 4 x 3.5 | 1.8 x 0.9 x 0.4 | 45 | 1.53 | 187 | 1.13 | SE | pT2N1M0 (II) |
| TGCT5 | 9 x 6 x 5.5 | 3.5 x 2.0 x 0.5 | 44 | 2 | ⇑612 | ⇑18.3 | SE | pT1NxM0 (I) |
| TGCT6 | 6.5 x 6.0 | 3.0 x 1.0 x 0.4 | 35 | ⇑117 | 192 | ⇑157 | NS, Mixed (EC - 60%*; YST - 30%*; TE - 10%) | pT2NxM0 (II) |
| TGCT7 | 4.5 x 3 x 2.7 | 1.6 x 1.0 x 0.4 | 27 | ⇑34 | ⇑366 | 1.3 | NS, Mixed (EC - 65%; SE - 35%*) | pT1bN0M0 (I) |
| TGCT8 | 7.4 x 4.0 x 2.7 | 2.0 x 1.5 x 0.4 | 31 | 2.3 | 123 | 2.25 | SE | pT2N1M0 (II) |
| TGCT9 | 4 x 3.5 | 3.0 x 1.5 x 0.5 | 23 | ⇑27.6 | 188 | ⇑97 | NS, Mixed (TE - 80%*; EC - 11%; YST - 9%*) | pT1N0M0 (I) |
| TGCT10 | 1.9 x 1.5 x 1 | 1.1 x 0.8 x 0.3 | 40 | 0.2 | 176 | ⇑7.49 | SE | pT1aN0M0 (I) |
| TGCT11 | 3.4 | 1.6 x 0.9 x 0.6 | 33 | 0.2 | 179 | 3.28 | NS, Mixed (SE - 80%*; EC - 18%; YST - 2%) | pT2N0M0 (I) |
| TGCT12 | 1.4 x 1 x 0.9 | 0.7 x 0.5 x 0.2 | 48 | 1.2 | 243 | 4.4 | SE | pT2N0M0 (I) |
| TGCT13 | 3.7 x 3.6 | 2.6 x 1.0 x 0.3 | 30 | 0.2 | 137 | 1.30 | Teratoma (TE) (primary) | pT1N0M0 (I) |
| TGCT14 | 3.7 x 2.3 | 1.3 x 1.1 x 0.8 | 26 | 0.2 | 218 | 1.13 | TE (retroperitoneal metastasis) | pT2N1M0 (II) |

**Supplementary Table 3:** List of primers used for RT-qPCR experiments.

| **Gene** | **Forward primer sequence (5' - 3')** | **Reverse primer sequence (5' - 3')** | **T_annealing_ (ºC)** |
| --- | --- | --- | --- |
| *PAX6* | CTGAGGAATCAGAGAAGACAGGC | ATGGAGCCAGATGTGAAGGAGG | 60 |
| *POU5F1* | TCCCTTCGCAAGCCCTCAT | TGACGGTGCAGGGCTCCGGGGAGGCCCCATC | 60 |
| *SOX2* | CCCCCGGCGGCAATAGCA | TCGGCGCCGGGGAGATACAT | 60 |
| *NANOG* | CTCCAACATCCTGAACCTCAGC | CGTCACACCATTGCTATTCTTCG | 60 |
| *LIN28* | GAAGCGCAGATCAAAAGGAG | CTGATGCTCTGGCAGAAGTG | 60 |
| *LIN28B* | GGAGCCCCTGTTTAGGAAGT | TCAAGGCCACCACAGTTGTA | 60 |
| *GUSB* | CTCATTTGGAATTTTGCCGATT | CCGAGTGAAGATCCCCTTTTTA | 60 |

**Supplementary Table 4:** List of antibodies used for the western blot experiments.

| **Antibody** | **Company** | **Dilution** | **Species** |
| --- | --- | --- | --- |
| Alix | Santa Cruz Biotechnology (sc-53540) | 1:300, 5% milk in TBS-T O.N., 4°C | Mouse |
| CD9 | Santa Cruz Biotechnology (sc-13118) | 1:500, 5% BSA in TBS-T O.N., 4°C | Mouse |
| CD81 | Santa Cruz Biotechnology (sc-166029) | 1:500, 5% BSA in TBS-T O.N., 4°C | Mouse |
| CD63 | Santa Cruz Biotechnology (sc-5275) | 1:300, 5% BSA in TBS-T O.N., 4°C | Mouse |
| TSG101 | Santa Cruz Biotechnology (sc- 7964) | 1:300, 5% BSA in TBS-T O.N., 4ºC | Mouse |
| HSC70 | Santa Cruz Biotechnology (sc-7298) | 1:500, 5% BSA in TBS-T O.N., 4ºC | Mouse |
| Cytochrome-c | Santa Cruz Biotechnology (sc-13560) | 1:500, 5% BSA in TBS-T O.N., 4°C | Mouse |
| Albumin | Santa Cruz Biotechnology (sc-271605) | 1:1000, 5% BSA in TBS-T O.N., 4ºC | Mouse |
| NANOG | Cell signaling (C52G3) | 1:500, 5% milk in TBS-T O.N., 4ºC | Rabbit |
| PAX6 | Invitrogen (13B10-1A10) | 1:500, 5% milk in TBS-T O.N., 4ºC | Rabbit |
| B-ACT | Sigma-Aldrich (#A1978) | 1:10000, 5%milk in TBS-T, 1h incubation, RT | Mouse |

**Figure S1:** Graphical representation of ATRA treatment schematic used for in the NT2 cell line.

**Figure S2:** Total number of particles/ml (A, B), and mode particle size (C, D) for all separate (T)GCT cell lines lEV and sEV NTA experiments. Data shown as mean ± SEM for 3 independent experiments.

**Figure S3:** RNA concentration measurement for the (T)GCT cell line-derived EV populations (A) and RNA concentration normalized to the number of particles: RNA per particle ratio (B). Data shown as mean ± SEM; * - p < 0.05.

**Figure S4:** Raw figures of the western blots performed for the cell and tissue-derived EVs. Red arrow pinpoint blots that were represented in Figure 2A.

**Figure S5**: Raw figures of the western blots performed for the plasma-derived EVs. Red arrow pinpoint blots that were represented in Figure 2B.

**Figure S6:** Representative bright field microscopy imaging of NT2 cells 5 and 10 days after treatment start with vehicle (A,B) and ATRA (C,D).

**Figure S7:** Western blot for pluripotency-related factors NANOG and PAX6, and for Beta-actin in vehicle and ATRA-treated NT2 cells.

**Figure S8:** Raw figures of the western blots performed for the ATRA-treated cells. Red arrow pinpoint blots that were represented in Figure S7.

**Figure S9:** Secretion range (NTA particles per cell ratio) for vehicle and ATRA-treated cells, in lEV (A) and sEV (B) populations. Data shown as mean ± SEM for 5 independent experiments; * - p < 0.05.

**Figure S10:** RNA concentration measurements in tumor tissue vs non-tumoral adjacent tissue, in lEV (A) and sEV (B) populations. Data shown as mean ± SEM for 5 independent experiments; ** - p < 0.01.

**Figure S11:** Tissue cellular levels for miR-371a-3p (A), miR-372-3p (B), miR-373-3p (C) and let-7e (D), in non-TE TGCT, TE and adjacent testicular parenchyma tissues. Data shown on a log scale as mean ± SEM; * - p < 0.05, *** - p < 0.001.

**Figure S12:** Spearman correlation analysis for tissue cellular and conditioned medium (CM) lEV and sEV microRNA levels (A – miR-371a-3p; B – miR-372-3p, C – miR-373-3p and D – let-7e).

**Figure S13:** Number of particles per sample for plasma-derived EVs in TGCT patients and healthy donors, lEV (A) and sEV (B) fractions. Data shown as mean ± SEM.

**Figure S14:** MiR-371a-3p (A), miR-372-3p (B), miR-373-3p (C) and let-7e (D) plasma circulating levels for non-TE TGCT, TE and healthy donors. Data shown as mean ± SEM; ** - p < 0.01, *** - p < 0.001.

**Figure S15:** Spearman correlation analysis for plasma circulating and EV-secreted (lEV and sEV) microRNA levels (A – miR-371a-3p; B – miR-372-3p, C – miR-373-3p and D – let-7e).
